# Use of Urban Health Indicator Tools by Built Environment Policy- and Decision-Makers: a Systematic Review and Narrative Synthesis

**DOI:** 10.1007/s11524-019-00378-w

**Published:** 2019-09-03

**Authors:** Helen Pineo, Ketevan Glonti, Harry Rutter, Nici Zimmermann, Paul Wilkinson, Michael Davies

**Affiliations:** 1 Institute for Environmental Design and Engineering, Bartlett School of Environment, Energy and Resources, University College London, Central House, 14 Upper Woburn Place, London, WC1H 0NN UK; 2 School of Humanities and Social Sciences, University of Split, Split, Croatia; 3 INSERM, U1153 Epidemiology and Biostatistics Sorbonne Paris Cité Research Center (CRESS), Methods of Therapeutic Evaluation of Chronic Diseases Team (METHODS), 75014 Paris, France; 4 Paris Descartes University, Sorbonne Paris Cité, Paris, France; 5 Department of Social & Policy Sciences, University of Bath, Claverton Down, Bath, UK; 6 Department of Social and Environmental Health Research, London School of Hygiene and Tropical Medicine, Keppel Street, London, WC1E 7HT UK

**Keywords:** Indicators, Indices, Evidence, Urban metrics, Urban policy, Urban planning, Built environment, Healthy cities, Social determinants of health

## Abstract

Global initiatives have raised awareness of the need for cross-departmental and cross-sectoral activities to support urban health, sustainability, and equity, with respective indicators routinely used as a way to catalyze and monitor action toward pre-defined goals. Despite the existence of at least 145 urban health indicator (UHI) tools globally, there has been very little research on the use of indicators by policy- and decision-makers; more attention has been devoted to their development and validation. This paper describes the second part of a two-part systematic review of the characteristics (part A) and use (part B, this part) of UHI tools by municipal built environment policy- and decision-makers. Part B is a narrative synthesis of studies on the use of UHI tools. This PRISMA-P compliant review follows a mixed methods sequential explanatory design. The search was conducted using seven bibliographic databases, grey literature searches, and key journal hand searches. Ten studies describing the use of ten UHI tools in seven countries were included in the narrative synthesis, resulting in development of a theory of change (ToC). We found that both expert-led and participatory indicator projects can be underpinned by research evidence and residents’ knowledge. Our findings contradict the dominant view of indicator use in policy-making as a linear process, highlighting a number of technical, organizational, political, knowledge, and contextual factors that affect their use. Participatory UHI tools with community involvement were generally more effective at supporting “health in all policies” and “whole-of-society” approaches to governing healthy cities than expert-led processes. UHI tool producers proposed a range of techniques to address urban health complexity characteristics. Finally, in combining data from both parts of the review, we found that potentially important UHI tool features, such as neighbourhood-scale data, were influential in the use of indicators by built environment policy- and decision-makers.

**Electronic supplementary material:**

The online version of this article (10.1007/s11524-019-00378-w) contains supplementary material, which is available to authorized users.

## Introduction

Global initiatives including the United Nation’s Sustainable Development Goals (SDGs) and the World Health Organization’s Healthy Cities Network have raised awareness of the need for cross-departmental and cross-sectoral activities to support urban health, sustainability, and equity [1, 2]. The establishment and use of relevant indicators, and in particular of urban health indicator (UHI) tools, is one route through which such initiatives seek to catalyze and monitor action toward these goals [3–5, 7]. We define UHI tools as “collection [s] of summary measures about the physical urban environment’s contribution to human health and wellbeing,” with a broad interpretation of health that expands to “related concepts of quality of life, liveability and wellbeing” [9]. Our previous study (called part A of this review) provides a global census and analysis of UHI tools and their characteristics (described below) [10]. We confirmed that attention has been devoted primarily to indicator development and validation, and there has been very little research on the use of such indicators by policy- and decision-makers [10–13, 15, 18].

Part A of our review extracted and analyzed data about the characteristics of 145 UHI tools, comprising 8006 indicators. We developed a taxonomy, classifying UHI tools by topic, spatial scale, format, scope, and purpose. UHI tools were produced in 28 countries, and a further 28 tools could be applied internationally. Our review supported the argument that neighbourhood-scale data are increasingly available and displayed on interactive maps, suggesting that such tools would be useful for urban planners, particularly to identify spatial and health inequalities [10, 19, 20]. We found a degree of similarity in the domains measured across UHI tool topics particularly among health and well-being, quality of life, and liveability. The majority of UHI tools in the review (82.8%, 120/145) intended to inform policy- and decision-making and were based on evidence (e.g., peer-reviewed studies underpinned 52.4% (76/145) of UHI tools).

UHI tools are often proposed for use by policy-makers assuming that a rational evidence-based policy model is in place, yet knowledge translation and policy scholars critique this model and the potential for indicators to be used in policy-making [12, 13, 18, 21, 22]. Innes and Booher claimed that most indicator reports fail to inform policy because their producers “relied on a simplistic model of how information drives policy” [12]. While Webster and Sanderson described WHO Healthy City Indicators as part of a logical “evidence-based, rational policy making and priority setting” process [23]. Given these diverse views, indicators have been variously conceptualized as rational technical tools in a linear policy process or as social constructs defined by local negotiation and context [11, 13, 15].

There are also diverse views about how indicators support policy- and decision-makers with the complexity of urban health. Complex systems are characterized as interconnected, dynamic, non-linear, adaptive, and governed by feedback, among other features [24–26]. The impact of urban environments on health has been described as an emergent property of a complex system composed of multiple subsystems such as housing, transport, and air quality [27–29]. Components of the built environment interact with social and economic factors, creating health equity challenges and adding further complexity [30]. This complexity hinders the study of urban environment exposures and effects, the production of validated indicators, and the creation of appropriate policy responses [5, 11, 28, 31, 32]. The first part of this review (part A) demonstrated that UHI tool producers are aware of these complexity challenges, yet few described the role of indicators in strategies to address them [10].

Notwithstanding the technical improvements to UHI tools identified in part A, there is a lack of research on whether they have succeeded in informing built environment policy- and decision-makers or attempted to support them with the complexity of urban health. To address this gap, this paper describes the second part (part B) of a two-part systematic review of the characteristics and use of urban health indicator (UHI) tools by municipal built environment policy- and decision-makers. Our narrative synthesis analyzes studies on the use and perceptions of these tools. Given the potential importance of the process of indicator development, as highlighted by Innes and Booher, we investigate the circumstances and actors involved in creating UHI tools and the impact of such processes [12].

## Methods

The review methods are outlined in the PRISMA-P compliant protocol with additional information in the part A results, both published previously [9, 10]. The review follows a mixed methods sequential explanatory design [33]. We combined quantitative data about the characteristics of UHI tools (part A) with qualitative data about their use (part B). The search strategy and review of papers were conducted simultaneously for both parts of our broader study; however, the review methods diverged with regard to eligibility criteria, quality appraisal, data extraction, and synthesis. The method for part B was informed by a scoping review and systematic reviews of evidence use by municipal policy-makers [9, 34, 35]. The Supplementary material of this paper contains additional information about definitions, search strategy, quality appraisal, narrative synthesis, thematic analysis, and development of a theory of change.

### Search Strategy

The search strategy was reported in the protocol and part A results and is thus only minimally discussed here with further information available in the Supplementary material, section 1.1.2 [9, 10]. The search was conducted from Jan 27, 2016 to Feb 24, 2016, using seven bibliographic databases, grey literature searches and key journal hand searches. Google Advanced searches were conducted on six urban planning practitioner and health promoting organizations’ websites and the Internet using specified search terms in line with the search strategy for databases. These websites were selected to find either UHI tools or studies about their use in municipal built environment policy-making, building on initial findings from the scoping review.

The search terms were identified through the scoping review and included key terms for (1) urban environment (e.g., urban, metropolitan, city, environment, neighbourhood, community), (2) health and related concepts (e.g., determinant, public, health, well-being, wellness, quality, livability) and (3) indicator (e.g., benchmark, tool, indicator, index, indices, measure, metric, profile, assessment, score, standard).

### Eligibility Criteria

Eligibility for part B required all of the following criteria to be met as described in the protocol:

- Reports substantive data on views, attitudes or knowledge about the use of an urban health indicator tool in the policy-making or decision-making process, or about the implementation of specific policies, interventions or programmes informed by these (modified from Lorenc et al.) [34]
- Includes policy and/or decision-makers from one of the following policy fields in local government: housing, transport, urban planning, and regeneration
- Reports qualitative or quantitative data
- Published in English (in any country) [9]

### Screening and Quality Appraisal

All documents were screened (in Eppi-Reviewer) by HP, and a random sample of 10% of documents were screened by KG at the title and abstract and full paper screening stages. Differences were resolved through discussion. A key point of discussion was whether studies reported substantive data which was interpreted on a case-by-case basis. The researchers looked for information that could be extracted and analyzed beyond a single sentence or paragraph. Studies included in part B were appraised using the UK National Institute for Health and Care Excellence (NICE) quality appraisal tool for qualitative studies [36]. The Supplementary material contains additional information and a copy of the completed quality appraisal checklists.

### Data Extraction and Synthesis

Studies that met the eligibility criteria for part B were included in a narrative synthesis, using the full text of the study for analysis. The narrative synthesis was informed by Popay et al. [37]. The synthesis was developed using textual descriptions, tabulation, semantic coding, thematic analysis, vote-counting as a descriptive tool and subgroup analysis. Data were analyzed using NVivo qualitative data analysis software (QSR International Pty Ltd., version 11.4.3, 2017). Based on Popay et al.’s guidance, data coding sought to inform, and was informed by, a theory of change (ToC) about what worked, for whom and in what circumstances. The ToC was developed iteratively and inductively (described in more detail in Supplementary material, section 1.1.6, including a completed checklist developed by Breuer et al. [38] for reporting ToC development). During inductive data analysis, we developed a distinction between UHI tool development processes as “expert-led” or “participatory” and categorized tools within these groups by comparing details on the UHI tool development process reported by UHI tool producers (see Supplementary material, section 1.1.7). Our primary criterion for distinguishing between expert-led and participatory development processes was whether or not a range of stakeholders were involved in selecting indicators.

## Results

**Fig. 1:**
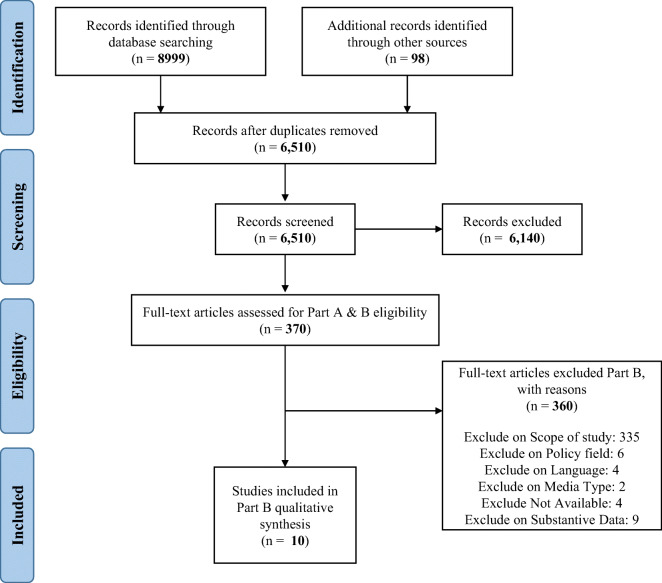
The flow of records in the review

Figure 1 shows the flow of records in the review. As in part A, 9097 records were identified from the bibliographic database, Internet, and journal searches. After duplicates were removed, 6510 titles and abstracts were screened, of which 370 were included in the full-text review. Finally, 10 studies were included in the part B narrative synthesis. A total of 360 studies were excluded on the basis of scope, policy field, language, media type, availability, or not reporting substantive data.

### Characteristics of Included Studies

Table 1 shows the characteristics of the 10 included studies and Table 2 describes the 10 UHI tools described in these studies. Most of the studies (7/10) were case study designs using a range of qualitative data collection methods including participant observation, interviews, document analysis and group discussions. With two exceptions, the studies were written by individuals involved in developing the UHI tool being investigated (for one of the exceptions, this could not be confirmed, the other was reported by independent academics). The majority of the UHI tools (8/10) were used in high income countries (the USA, Australia, the UK, and Belgium) and two were used in lower middle-income countries (India and Kenya). One study gathered perceptions on the potential value of UHI tools in South Africa. Most of the studies were conducted relatively recently (two between 1988 and 2000 and eight between 2008 and 2015). All of the studies addressed the urban planning policy field.

**Table 1:** Description of studies included in narrative synthesis

Authors and year	Country	UHI tools investigated	Study type and data collection methods	Policy field(s)	Authors developed UHI tool?
Bhatia (2014) [39]	USA	San Francisco Indicators Project	Case study: author’s experience and observations	Urban planning, transport, community development	Yes
Corburn and Cohen (2012) [30]	USA and Kenya	Richmond Health and Wellness Element Indicators and Urban Health Equity Indicators for Mathare Informal Settlement	Case studies (2): authors’ experiences working collaboratively with communities and local agencies to develop the UHI tools	Urban planning	Yes
Corburn et al. (2014) [46]	USA	Richmond Health Equity Indicators (aka Healthy City Diamonds)	Case study: participant observation, interviews, and document analysis	Urban planning, neighbourhood safety, public works	Yes
Farhang et al. (2008) [40]	USA	San Francisco Indicators Project	Case study: not stated	Urban planning (and other “city agencies”)	Yes
Hunt and Lewin (2000) [44]	India and South Africa	Core Environmental Health Indicators in Lucknow and Calcutta	Case studies (2): interviews, observation, and group discussions	Urban planning and environmental services	Yes
Landis and Sawicki (1988) [6]	USA	Places Rated Almanac	Mixed methods: interviews and surveys	Urban planning	No
Lerman (2011) [47]	USA	(Seattle) Healthy Living Assessment	Project report: not stated	Urban planning (specifically neighbourhood planning)	Yes
Lowe et al. (2015) [8]	Australia	Community Indicators Victoria (and other non-specified indicators)	Workshops	Urban planning (and other non-specified government policy-makers)	Yes
Shepherd and McMahon (2009) [45]	England	(Bristol) Quality of Life Indicators	Case study: interviews	Urban planning, transport, regeneration, officers working with Local Strategic Partnership, sustainable development	Unknown
Van Assche et al. (2010) [41]	Belgium	Flemish City Monitor	Case study: authors’ experience working with 13 Flemish cities in developing and reporting the UHI tool	Urban planning (and other non-specified government policy-makers)	Yes

**Table 2:** Development process and characteristics of the UHI tools investigated by included studies (NBHD: neighbourhood)

Tool/Index	Development of UHI tool			CHARACTERISTICS	
	Lead organisation type	Development process	Evidence informed UHI tool	Mapping function	Simplified Scale
(Bristol) Quality of Life Indicators [45]	City Government	Expert led	Unknown	Yes	City & NBHD
Community Indicators Victoria [8]	Research Institution		Peer-reviewed literature	Yes	City & larger
Places Rated Almanac [6]	Private Sector		Unknown	Yes (static)	City
(Seattle) Healthy Living Assessment (HLA) [47]	City Planning Dept.		Peer-reviewed literature	No	NBHD
Core Environmental Health Indicators in Lucknow and Calcutta [42]	Research Institution	Participatory	Unknown (Community derived)	No	NBHD
Flemish City Monitor [41]	Research Institution		Peer-reviewed literature	No	City
Richmond Health and Wellness Element Indicators [30]	City Government		Peer-reviewed literature	No	City & NBHD
Richmond Health Equity Indicators (aka Healthy City Diamonds) [46]	Not-for-Profit Collaboration		Community and expert input	No	City
San Francisco Indicator Project (SFIP) [39, 40]	City Public Health Dept.		Peer-reviewed literature	Yes	City & NBHD
Urban Health Equity Indicators for Mathare Informal Settlement [30]	Research Institution		Peer-reviewed literature	No	NBHD

### UHI Tool Development

The approach to developing and applying UHI tools, either expert-led or participatory, influenced the value and use of UHI tools. Therefore, a distinction is made between these two approaches when analyzing data and reporting results.

Expert-led UHI tools (4/10) generally followed a technical approach to indicator development, with a focus on environmental health exposures and outcomes (Table 2). Such UHI tools were developed to measure, compare, and assess the urban environment impact on health through translation of research evidence, with recognition of an evidence hierarchy. The community (i.e., the general public) was not central to the development of such tools, although their views may have been incorporated in some way, such as to inform UHI tool domains. Expert-led approaches sometimes involved an iterative process informed by engagement with local government (and other) stakeholders.

In contrast, participatory UHI tools (6/10) were described as emerging from a process of co-production with the community which placed relatively less importance on the hierarchy of evidence defining or validating indicators (Table 2). These participatory processes encouraged a wide range of city stakeholders, including citizens, local government officials, and academic experts to co-define concepts and co-produce indicators through an iterative process of local negotiation, determined by context. Some of these projects involved co-creation of policy and co-monitoring of policy impacts.

The expert-led and participatory characterizations are not absolute and variations among UHI tool processes are recognized. For example, experts technically led the Flemish City Monitor and the San Francisco Indicators Project (SFIP) development, yet multiple stakeholders, including the community, were significantly involved and made fundamental decisions about the process and indicators [39–41]. As a result, these projects are viewed as participatory in this study. In contrast, there was engagement with community organizations and government stakeholders to establish indicator domains for Community Indicators Victoria (CIV), but the majority of indicator selection and application was expert-led [42, 43].

### Uses and Benefits of UHI Tools

The development and application of UHI tools resulted in a number of benefits that had the potential to improve the built environment to promote health and well-being. Table 3 shows how many studies reported each use/benefit and whether these were achieved through UHI tools characterized by the following: (1) expert-led or participatory approaches and (2) indicator data presented at neighbourhood or city scale. The three uses and benefits outlined below are among the top four from Table 3. These were achieved by expert-led and participatory UHI tool approaches. The benefit of collaboration across stakeholders is discussed in the next section, which led to multiple outcomes beyond those described in Table 3.

**Table 3:** Reported uses and benefits from developing or applying UHI tools by development approach and spatial scale of indicator data. NBHD: neighbourhood

Uses and benefits of developing or applying UHI tools	Proportion Of UHI tools with this outcome									
	All UHI tools		Expert-led		Participatory		NBHD scale		City scale	
	*n*/10	%	*n*/4	%	*n*/6	%	*n*/6	%	*n*/4	%
Informed policy development	8/10	80	4/4	100	4/6	67	4/6	67	4/4	100
Created awareness and knowledge of urban health issues	8/10	80	2/4	50	6/6	100	6/6	100	2/4	50
Facilitated collaboration across stakeholders	7/10	70	4/4	100	3/6	50	4/6	67	3/4	75
Supported monitoring	7/10	70	3/4	75	4/6	67	5/6	83	2/4	50
Provided evidence of health or spatial inequalities	6/10	60	3/4	75	3/6	50	5/6	83	1/4	25
Identified local issues	5/10	50	3/4	75	2/6	33	4/6	67	1/4	25
Supported policy area prioritization	5/10	50	3/4	75	2/6	33	4/6	67	1/4	25
Defined urban health concept	5/10	50	3/4	75	2/6	33	4/6	67	1/4	25
Enabled public accountability through transparency of data	5/10	50	1/4	25	4/6	67	4/6	67	1/4	25
Supported lobbying for policy, action or funding	4/10	40	1/4	25	3/6	50	3/6	50	1/4	25
Resulted in policies/programmes which improve or protect the environment	4/10	40	2/4	50	2/6	33	4/6	67	0/4	0
Engaged the public or changed the public’s behavior	4/10	40	3/4	75	1/6	17	3/6	50	1/4	25
Promoted ownership of health issues by planning and other city departments	4/10	40	2/4	50	2/6	33	4/6	67	0/4	0
Highlighted community needs to local government	3/10	30	1/4	25	2/6	33	3/6	50	0/4	0
Supported performance management of city policy and decisions over time	3/10	30	1/4	25	2/6	33	2/6	33	1/4	25
Engaged politicians	3/10	30	2/4	50	1/6	17	2/6	33	1/4	25
Aided communication	3/10	30	1/4	25	2/6	33	2/6	33	1/4	25
Justified policies or decisions being taken by local government	2/10	20	1/4	25	1/6	17	2/6	33	0/4	0
Informed planning decisions or development proposals	2/10	20	1/4	25	1/6	17	2/6	33	0/4	0
Informed decisions about funding allocation	2/10	20	1/4	25	1/6	17	2/6	33	0/4	0
Facilitated benchmarking across communities or time	2/10	20	2/4	50	0/6	0	1/6	17	1/4	25
Improved capacity (knowledge/ability) in local government	1/10	10	1/4	25	0/6	0	1/6	17	0/4	0
Supported site selection for development	1/10	10	0/4	0	1/6	17	1/6	17	0/4	0

Informing policy development was the most widely noted benefit of developing and applying UHI tools. For example, in San Francisco, the UHI tool improved understanding of air quality issues in certain neighbourhoods within the city, which then led to specific policies to reduce ingress of polluted air into new housing [39]. Through dialogue between the city’s planning and public health departments, the public health team were able to understand the planners’ constraints and make appropriate recommendations to reduce the impact of air pollution in new housing.

UHI tools increased community and local government knowledge and capacity to improve urban health. In Cape Town, Lucknow and Calcutta study participants identified a virtuous cycle of community involvement in indicators to raise awareness and thereby improve city services [44]. In Bristol, knowledge gained by the community was described as “one of the most important outcomes” although it was “more of an unintended consequence” [45]. In San Francisco, indicators helped “citizens to participate more knowledgeably in decisions” [39] and “unequivocally increased Council member understanding of how human health is impacted by development” [40].

Monitoring was consistently described as a valuable function of UHI tools, as either a task for local government officials or a participatory governance process. The latter was promoted by Corburn and Cohen as part of an adaptive management process [30]. UHI tools with longitudinal data allowed local officials to observe trends and act early when problems arose. Monitoring through Bristol’s UHI tool “improved the targeting of investment in graffiti removal to prioritise Neighbourhood Renewal Areas which then quickly saw positive impacts on public perception” [45].

### Benefits of Community Involvement in UHI Tools

Involving communities in developing or using UHI tools resulted in additional outcomes compared to what could be achieved without their involvement. These could be achieved through either expert-led or participatory processes, although the latter were likely to have greater community involvement. There were four key benefits of community involvement in UHI tool development.

First, community involvement led to increased participation and sense of power in urban governance. Corburn et al. described the outcome of workshops with the community and city staff as being “crucial for generating policy solutions and transforming the governance relationships between the city and its residents” [46]. The indicator process in San Francisco “promote[d] meaningful public involvement in land use policy making by making explicit competing interests and facilitating consensus” [40]. Expert-led UHI tools also facilitated community involvement in planning processes. For example, through using Seattle’s HLA, “[c]ommunity members were engaged in the planning process in a genuine and productive way” [47].

Second, community input in UHI tools increased balancing of expert and lay knowledge claims and representation of community needs to policy-makers. Diverse stakeholders brought their own knowledge and priorities to UHI tool development. Project leaders determined how different perspectives and knowledge claims should be elicited and treated when developing indicators. For example, in San Francisco, “the quality of participation likely enhanced the interpretability, meaning, and relevance of indicators for stakeholders and contributed to the indicators’ usefulness in supporting stakeholders’ demands in the process of policy making” [39]. A wide range of stakeholders were consulted, contributed views, and made decisions, with the public health department acting as a final arbiter to interpret this range of information and apply indicator results with the city’s planning department.

Third, community participation in UHI tools created or exposed tensions between stakeholders which opened opportunities to negotiate solutions and build consensus. Tensions were related to conflicting views on how environment, land use, and health issues should be addressed through built environment policies. They were also related to power imbalances among actors and differing views on how indicators would be used to redress these. In Lucknow and Calcutta, the indicator development process created a positive opportunity to discuss issues and gain new perspectives about pre-existing tensions between residents, planners, and service providers. The process of developing indicators created opportunities for residents and government representatives to move beyond “stereotypic views” and “discuss common concerns … to improve the understanding of each other’s needs and constraints” [44]. This new shared understanding was found to have improved dialogue and thereby improved service delivery [44]. A very similar story was relayed about SFIP where the UHI tool was purposefully developed in the context of existing conflict to “foster dialogue among diverse stakeholders to help bridge the multiple and often competing interests placing demands on development” [40]. However, some stakeholders felt that SFIP was “stacked against development interests” and would therefore be used in attempts to stop new development [40].

Finally, the development and use of UHI tools with community members improved the knowledge of residents and city agencies about the varied environmental causes of health impacts, leading to examples of “health in all policies” and “whole-of-society” approaches, as defined by Kickbusch and Gleicher [48]. For example, SFIP increased knowledge and led some participants “to apply public health arguments and evidence in public policy dialogues on housing, economic, and environmental issues,” essentially resulting in a health in all policies approach [40]. In relation to whole-of-society approaches, in Seattle, the focus on residents’ views provided through application of the UHI tool “led to the inclusion of more actions that lie outside the realm of city departments” [47].

### Facilitators and Barriers of UHI Tool Development and Use

There were a number of facilitators and barriers to both developing and applying UHI tools that affected their ability to influence policy- and decision-making (Table 4). The facilitators and barriers spanned technical, political, knowledge, and organizational factors. Facilitators listed under a particular heading in Table 4 (e.g., knowledge) may have helped to overcome barriers of that same type or other types (e.g., political). These facilitators and barriers are further explained through the theory of change.

**Table 4:** Facilitators and barriers to applying (A) or developing (D) UHI tools

Facilitators	Type	Barriers
Data related to policy (A)	Technical	Not related to relevant policy or policy area (A)
Data measures of policy inputs and outputs (A)		Lacked new information/or adequate information (A)
Data available at small geographic scales and is comparable (A)		Inappropriate scale of data availability (D/A)
Data not expensive to obtain (D)		Data availability and cost of obtaining data (D/A)
Indicators include social and built environment elements (A)		Limited relevance of indicators to specific users (A)
Provides evidence to support advocacy (A)		Variation in how indicators are prioritized by different groups (D/A)
Measures public service performance (A)		Data did not match the population affected by new development (A)
Data collected over a long period (A)		
City managers receptive to indicator data (A)	Political	Politicians’ concern that indicators would reveal negative issues (A)
Indicator work is embedded in a local government department with influence over relevant policy or other departments (A)		Concern that indicators would be used to stop development (A)
		Concern that UHI tool would be used to create new regulations (A)
		UHI tool not accepted/valued by all stakeholders (A)
		Conflict between UHI tool stakeholders (A)
		Indicator outputs not politically or financially feasible (A)
		Complexity of policy-making process (A)
		Local leaders did not want policy advice from indicators (D/A)
Diverse knowledge incorporated via broad participation (D/A)	Knowledge	Knowledge gap about health and land-use (D)
Indicators are perceived as “neutral” or “objective” (A)		Knowledge gap about creation and application of indicators (D/A)
		Knowledge gap about translating indicator data into development plan recommendations (A)
Residents/citizens are involved in selecting indicators (D/A)	Organizational	Conflict or disagreement within the indicator producer group (D/A)
Indicator developer (or owner) is embedded in local authority (A)		Stakeholder availability and “permission” to participate (D)
Indicator data is integrated early in the planning process (A)		Limited agency/power of the indicator producer or users (D/A)
		Difficulty finding neutral space for all stakeholders to meet (D)
		Focusing stakeholder involvement away from grievances (D)
		Lack of collaboration across municipal departments (A)
		Not all stakeholders equally interested in producing indicators (D)
		Resource constraints (A)

### Relations between Characteristics and Use of UHI Tools

Several insights were identified by combining quantitative and qualitative data from parts A and B. First, a number of UHI tool uses and benefits were more commonly achieved through UHI tools which measured data at the neighbourhood and city-scale, compared to those which only measured city-scale data (see below). Second, despite the large number of UHI tools which mapped data spatially (64/145 [44.1%] in part A and 4/10 in part B) and the supposed benefits of presenting data on maps in the literature, using maps was not frequently mentioned in the studies of UHI tool development and application. Third, only one UHI tool (Places Rated Almanac) reported data through an index, or composite indicator, and the study concluded that it was not useful for built environment policy- and decision-making. See the Supplementary material for further details.

Of the part B UHI tools, 60% (6/10) measured data at the neighbourhood (and city) scale, compared to 59.3% (86/145 tools) of the part A UHI tools. Some outcomes of developing and applying UHI tools were more frequently achieved by the neighbourhood scale tools than the city scale tools, such as creating awareness of urban health issues, supporting monitoring and providing evidence of health or spatial inequalities (Table 3).

### Addressing Complexity with UHI Tools

Complexity was recognized as a feature of both policy-making and urban health systems with several examples of how the use of UHI tools may address these challenges. Two studies provided specific solutions, including an adaptive management approach and underpinning UHI tools with a normative or systems framework (described in the Supplementary material) [30, 41]. Based on their experience in India and South Africa, Hunt and Lewin were not convinced that UHI tools could influence the “complexity of the policy process,” identifying political and economic constraints as key barriers [44].

Appendix Table 6 outlines seven characteristics of complexity in urban health systems identified in the academic literature (adapted from Pineo et al. [11]) and summarizes potential solutions identified in the systematic review (parts A and B) and from the authors (marked by an asterisk).

### Theory of Change

**Fig. 2:**
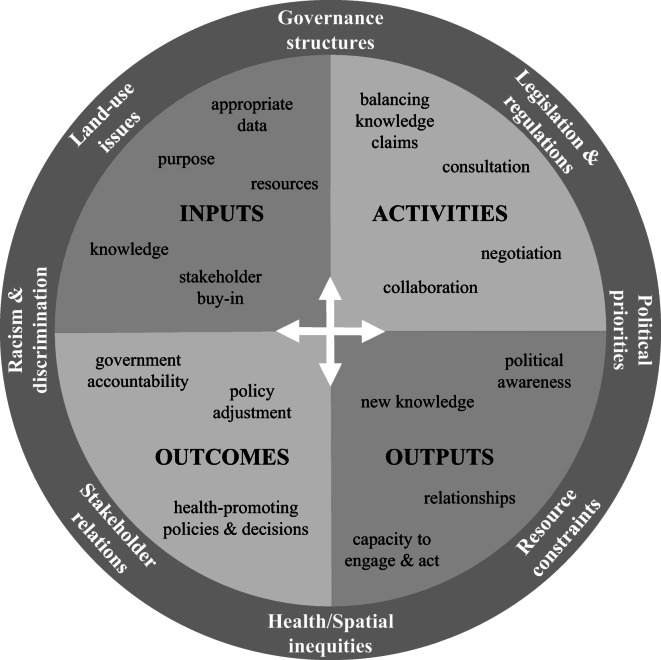
High-level visual summary of our ToC

Figure 2 is a high-level visual summary of our ToC. The visual shows four quadrants for inputs, activities, outputs and outcomes which are not necessarily sequential (i.e., occurring clockwise). Each quadrant contains key characteristics rather than a comprehensive description. The quadrants are affected by the external ring of contextual factors. Table 5 provides more detail about the ToC, differentiating between participatory and expert-led UHI tools. Many factors were common to both approaches, such as the requirement of resources and data. However, the importance and function of inputs and activities varied across the approaches. Furthermore, participatory approaches more often had the crucial difference of involving the community alongside a wide group of stakeholders which required different inputs (such as places to meet and buy-in from stakeholders) and activities (such as balancing competing knowledge claims and negotiating pre-existing conflicts or tensions).

Both participatory and expert-led UHI tool processes shared outputs such as increasing stakeholder knowledge. However, participatory processes (typically with greater community involvement) resulted in additional outputs such as a wider group of stakeholders gaining and applying new knowledge of urban health issues across multiple policies and activities (health in all policies and whole-of-society approaches), increased collaboration and communication among stakeholders, and policies that more directly responded to residents’ needs.

**Table 5:** Detailed theory of change of UHI tools influence on policy- and decision-making

Context	Approach	UHI tool development		UHI tool application	
		Inputs	Activities	Outputs	Outcomes
Governance structures, legislation and regulation, political priorities, resource constraints, health/spatial inequalities, stakeholder relations, racism and discrimination, land use issues	Participatory (with an emphasis on community involvement)	Resources for wide stakeholder involvement Places to meet Buy-in and permission to participate Wide stakeholder knowledge	Balance competing knowledge claims Negotiate pre-existing conflicts or tensions	City officials and residents gained new knowledge New knowledge applied to wide range of city activities and policies by all stakeholders Stakeholders gained mutual appreciation of constraints and opportunities Increased collaboration and new relationships across stakeholder groups Residents empowered to take further action Improved communication among stakeholders	Adopted policies to improve urban health through built environment which respond to residents’ (and other stakeholders’) needs City-wide activities and policies address urban health challenges
	Shared	Resources for data collection (over time) and analysis Appropriate data Identified indicator user	Link indicators to policy Underpin indicators with urban health research evidence	New knowledge about urban health, inequalities and priorities Increased awareness and political importance of urban health issues Indicator users monitor government performance Stakeholders use data to lobby for policy, action or funding Decision-makers use data to justify city policies or decisions	Built environment decisions support urban health objectives New development is designed to promote urban health Urban environment is monitored over time and policies are adjusted Residents or city stakeholders hold government to account
	Expert-led	Expert knowledge	Involve relevant indicator users Consult community in indicator development		Adopted policies to improve urban health through built environment

## Discussion

This study has contributed new knowledge about the use of UHI tools using a mixed methods systematic review. First, we found that UHI tools in our sample were developed using a combination of research evidence and residents’ knowledge and represented a middle ground between opposing epistemological characterizations of indicators as either rational tools or socially constructed artefacts. Second, our findings contradict the dominant view of indicator use in policy-making as a linear process, identifying a range of technical, political, knowledge, organizational and contextual factors that impact UHI tool use (shown through the ToC). Third, participatory processes of UHI tool development brought about useful outcomes for urban environment policy- and decision-makers; however, this was not UHI tools’ only path of influence. Fourth, community involvement in UHI tools (typically achieved by participatory approaches) resulted in uses and benefits that would support health in all policies and whole-of-society approaches to governing healthy cities, such as creating distributed awareness and knowledge of urban health issues. Fifth, UHI tool producers proposed a range of techniques to address urban health complexity characteristics; however, some were cautious as to whether such methods can influence the complexity of policy- and decision-making. Finally, in combining data from parts A and B, the review has shown that potentially important UHI tool features, such as neighbourhood-scale data, were influential in the use of indicators by built environment policy- and decision-makers. Our findings support UHI tool producers with better understanding of how indicators influence policy (e.g., through the ToC) which could shape future UHI tool development and improve their impact.

We believe this is the first narrative synthesis of studies on the use of UHI tools, the first study on this topic to use the sequential explanatory mixed methods design and the first ToC of the use and benefits of UHI tools. We have contributed new synthesized knowledge on what works, for whom, and in what circumstances. The protocol was published prior to conducting the review and followed best practice procedures for systematic review design and reporting [9, 49, 50]. The synthesis procedure followed best practice guidance [37]. The review covered a range of income settings; however, studies were predominately in high income settings.

The review was limited to English language publications, potentially excluding UHI tools from non-English language countries. The included studies were primarily case studies (7/10) conducted by the same individuals who developed the UHI tools^1^ and therefore may have overemphasized positive benefits of using indicators. The study designs were heterogeneous, and there were few available studies. One included report was not a peer-reviewed journal paper. In conducting this review, all studies and reports which met the eligibility criteria were included, regardless of methods and risk of bias. Our ToC is only representative of the included studies and could be improved through wider consultation with indicator producers and users.

Our narrative synthesis identified a middle ground for debates about the epistemological basis of indicators. UHI tool producers combined rationalist and constructivist approaches to indicator selection. Evidence-based indicators (i.e., scientific evidence) were supported and desired by all stakeholders. Regardless of the type of knowledge claim underpinning UHI tools, some stakeholders were suspicious of the use of such metrics for justifying built environment policies and decisions. For example, some politicians and developers argued against indicators which could be used to block (economic) development [40, 41]. Given the potential value of UHI tools in representing community interests in planning and development, particularly those related to equity, it is essential to understand how these interests may be subordinated to more powerful actors in urban governance. Future research is needed to explore the weight of community-informed indicators in decision-making.

We have previously discussed whether similarities across UHI tools supported an argument for greater indicator standardization to reduce duplication of research efforts [10]. In relation to sustainability and social indicators, scholars have argued that the role of indicators within governance processes and the process of developing indicators is equally or more important than the resulting indicator data [12, 13]. Our ToC shows the value of participatory processes and contradicts the dominant view of indicator use as a linear process. A number of factors spanning technical, organizational, political, and knowledge (and wider context) were influential in determining whether indicators could support policy- and decision-making. The processes of indicator development and application with diverse stakeholders (including the community) were integral to achieving benefits such as negotiation and consensus-building, balancing knowledge claims, supporting health in all policies and whole-of-society approaches, community participation, and local learning. Standardization would potentially risk the achievement of such benefits. However, we support our previous assertion that further research could identify whether a global set of evidence-based urban health indicators could be a starting point for local efforts, which would continue through a process of local prioritization and application of indicators using local data [10].

Community representatives sought to empower themselves by using UHI tools as a mechanism to exert influence in urban governance (such as to resist development or argue for funding) on the basis of health-related arguments. However, UHI tools were not necessarily viewed by some developers and politicians as a legitimate input to policy- and decision-making [40, 41]. The validity of UHI tools appears to have been contested in settings where stakeholders felt that existing powers and governance mechanisms were at risk of being disrupted through the use of indicators. The Pastille Consortium argued that conflict among actors reduced the likelihood that indicators would inform policy [51]. However, Innes and Booher recognize the likelihood of conflict in complex urban governance challenges and the value of raising diverse views to collaboratively develop solutions [21]. Our review found cases where UHI tool processes were used to reduce conflict among actors by creating opportunities for discussion and negotiation that would not have happened otherwise. The role of UHI tools in supporting diverse actors to address power imbalances in urban governance is an area for further research.

The simplification and communication of complex phenomena are often cited as key benefits of indicators, although scholars have pointed to the potential risks for policy-making including political manipulation of indicators and inappropriate policy responses [30, 52–54]. The review (parts A and B) identified a number of strategies in indicator development and application that may support policy- and decision-makers with complexity. However, some study authors evaluated in this review felt that UHI tools may not be effective in influencing the complex policy- and decision-making process [41, 44]. Further research is required to investigate the value of UHI tools in relation to simplifying, representing or addressing complexity in urban health and policy-making.

### Electronic Supplementary Material

ESM 1 (DOCX 185 kb)

## Appendix

**Table 6:** Characteristics of complexity in urban health systems (adapted from Pineo et al., 2018) [11] and proposed characteristics of UHI tools which could help address complexity

Characteristic	Description (in urban health terms)	Example for urban health system	How could UHI tool characteristics help address this challenge?	How could UHI tools help address this challenge in policy- and decision-making?
Dynamic [25]	Health and well-being impacts and/or exposures change over time (possibly in unpredictable ways)	E.g., Air pollution has long-term trends (increasing over time), seasonal trends and extremes (spikes).	Monitoring urban environment exposures and outcomes over time. [5, 30]	• Involving multiple stakeholders and the community in a process of “adaptive management” including co-design of indicators and policy and co-monitoring of impacts and policy adjustments. [30] • Using cross-departmental and multi-stakeholder UHI tool development processes to identify and discuss interconnections and policy responses. [30] • Providing policy- and decision-makers with indicators which measure and monitor multiple factors across policy areas. [30, 41, 45] • Making the components of urban health and liveability (and interconnections between these) explicit to decision-makers through a normative or systems framework underpinning indicators. [3, 41]
Number of elements [14, 26]	High number of variables within system	E.g., Transport system includes many elements which interact to create effects such as a walkable community.	Including a large number of indicators of exposures and outcomes. [16, 18]	
Interconnected [25]	Multiple interactions across and within systems	E.g., Transport emissions affect health through air pollution while contributing to climate change which has additional health impacts.	Including quantitative and qualitative data to provide a holistic picture. [30] Including measures of known interactions across indicators or domains (e.g., transport and air quality) and making known interactions explicit in reporting.*	
Non-linear structure [25]	Non-linear relationship between exposure and health and well-being impact—effects are rarely proportional to causes	E.g., Impact of vehicle speed on pedestrian injury/death does not change proportionately as speed increases.	Reporting clear thresholds or tipping points within or alongside indicators.*	
Feedback [25]	System elements interact recursively (in feedback loops) to change the behavior of the system	E.g., Increasing road capacity usually has the unintended effect of increasing traffic congestion by attracting more drivers.	Reporting links between indicators with description of feedback loops.*	
Counter-intuitive [25]	Health and well-being impacts are distant in space and time to exposures	E.g., The presence of many fast food outlets in a community may result in increased obesity levels over time.	Ensuring exposures and outcomes are measured at appropriate spatial scales and longitudinally. Making delays explicit in reporting.*	
Emergent behavior [14, 26]	Health and well-being effects are greater than the sum of individual effects within the system	E.g., A park or 20-mph limit are not sufficient on their own to support physical activity, but are effective if combined with other elements (such as pavements, mixed land uses).	Reporting data which form part of an urban health system (behavior, outcome or exposure, e.g., physical activity), crossing typical UHI tool domains.*	

## Footnotes

1 We could not confirm whether Shepherd and McMahon [45] (one out of seven case studies) were involved in developing the (Bristol) quality of life indicators.
