# Supplementary material for: Use of Urban Health Indicator Tools by Built Environment Policy- and Decision-Makers: a Systematic Review and Narrative Synthesis
Source: J Urban Health. 2019 Sep 3;97(3):418–35. doi: 10.1007/s11524-019-00378-w (PMC7305281; doi:10.1007/s11524-019-00378-w)
Supplement: Supplementary file 1 — (DOCX 185 kb) [file 11524_2019_378_MOESM1_ESM.docx]

**Supplementary Material for ‘Use of urban health indicator tools by built environment policy and decision-makers: a systematic review and narrative synthesis’**

Helen Pineo, Ketevan Glonti, Harry Rutter, Nici Zimmermann, Paul Wilkinson, Michael Davies

Table of Contents

[1.1 Additional methods 1](#_Toc13556398)

[1.1.1 Definitions 1](#_Toc13556399)

[1.1.2 Search strategy 1](#_Toc13556400)

[1.1.3 Quality appraisal 2](#_Toc13556401)

[1.1.4 Data extraction 2](#_Toc13556402)

[1.1.5 Narrative synthesis and thematic analysis 2](#_Toc13556403)

[1.1.6 Theory of Change 3](#_Toc13556404)

[1.1.7 Expert-led and participatory development processes 9](#_Toc13556405)

[1.2 Relations between characteristics and use of UHI tools 14](#_Toc13556406)

[1.3 UHI tool approaches to address complexity 15](#_Toc13556407)

[1.4 Completed quality appraisal checklists 16](#_Toc13556408)

[References 36](#_Toc13556409)

# 1.1 Additional methods

In addition to the methods described in the main paper, here we describe definitions, search strategy, quality appraisal, narrative synthesis, thematic analysis and development of the theory of change.

## 1.1.1 Definitions

UHI tools were defined in the protocol as ‘a collection of summary measures about the physical urban environment’s contribution to human health and wellbeing.’^1^ The definition includes the health-related concepts of quality of life (QOL), liveability, wellbeing (defined by Pineo et al.) and walkability/physical activity.^2^

## 1.1.2 Search strategy

As reported in the Part A results, Table 1 shows the databases, websites and hand-searched journals. Websites and journals were selected on the basis of the scoping review. In other words, these websites/journals were sources of UHI tools through an initial Google search (scoping review), and thus a more thorough search was applied during the full search. There were no date restrictions for searches of the bibliographic databases and websites. Date restrictions for the hand searching of key journals were between one to five years, depending on the relevance of articles found and the number of volumes per year

**Table 1 Databases, websites and journals searched for the review, including years hand searched for the journals**

| **Source type** | **Source** |
| --- | --- |
| Bibliographic Databases | Applied Social Sciences Index and Abstracts (ASSIA)  Campbell Library  Embase  Medline  Scopus  Social Policy and Practice  Web of Science Core Collection (includes the Social Sciences Citation Index) |
| Websites | Town and Country Planning Association (UK)  Royal Town Planning Institute (UK)  Planning Institute of Australia  American Planning Association  Built Environment and Public Health Clearinghouse (USA)  World Health Organization Europe, Urban Health, Healthy Cities |
| Hand-searched Journals | Annual Review of Public Health (5 years)  Social Science and Medicine (3 years)  BMC Public Health (1 year)  Social Indicators Research (3 years) |

## 1.1.3 Quality appraisal

Studies included in Part B were appraised using the UK National Institute for Health and Care Excellence (NICE) quality appraisal tool for qualitative studies.^3^ One study reported quantitative data (from a survey) which was conducted via interviews. Therefore, the qualitative study appraisal tool was applied. The studies in this review were heterogeneous regarding study design and reporting of methods and results. None of the studies were rejected on the basis of methodological quality, nor was the analysis differentiated with regard to study robustness. The completed quality appraisal checklists are in section 1.4.

## 1.1.4 Data extraction

Data about each study’s characteristics were extracted (e.g. author, year, country) as shown in the protocol.^1^ These data were contextual and did not form part of the narrative synthesis (apart from the UHI tool).

## 1.1.5 Narrative synthesis and thematic analysis

The synthesis process was iterative. It began with familiarisation with the included studies and producing a written descriptive summary of the studies. Then semantic coding and interpretation were conducted using thematic analysis. A sub-group analysis was undertaken using data about UHI tool characteristics from part A, for example examining benefits of UHI tools based on different scales of data aggregation.

The thematic analysis used a hybrid inductive and deductive coding approach based on Fereday and Muir-Cochrane’s approach.^4^ Deductive coding used an *a priori* codebook (see below), while inductive coding involved identification of ‘an important moment’ in the data and ‘encoding it prior to interpretation.’^4^ Based on Braun and Clarke’s distinction of latent and semantic level coding, data were coded at the semantic level.^5^ In other words the ‘explicit or surface meanings of the data’ were used, rather than looking ‘*beyond* [sic] what a participant has said or what has been written.’^5^

The codebook consisted of five code categories based on the study’s objectives (set out in the protocol) and the guidance by Popay et al.^1,6^ The full code set was updated iteratively as new factors were identified. The codebook categories allowed for the inductive development of additional codes to describe more detail within the categories, which included:

1. Theory of change: short descriptions of what worked, for whom and in what circumstances
2. Facilitators: factors that aided the use of UHI tools
3. Barriers: factors that hindered the use of UHI tools
4. Uses or benefits: built environment policy and decision-makers’ perceptions of the uses and benefits of developing and applying UHI tools
5. Complexity: the complex nature of indicator development, urban health issues and application of indicators in policy and decision-making.

The coded text was iteratively grouped and explored through tabulation and concept mapping to produce the narrative synthesis of studies. For example, text which were deductively coded as ‘facilitators’ were inductively coded with more detail such as ‘presentation of information’ and ‘data quality’. All of the data coded as ‘facilitators’ were tabulated and re-grouped under types of facilitators based on descriptions from the studies. The same process was followed for other categories such as ‘barriers’ and ‘uses or benefits’.

The interpretation of qualitative data was aided by the background literature from the scoping review and the theoretical lenses of communicative planning, collaborative rationality and systems thinking.^7–9^ For example, community participation and perceptions emerged as important contextual factors in the use of UHI tools by urban planners through the inductive coding process. Interpretation of this data with background literature and communicative planning and collaborative rationality theoretical lenses, helped to put community participation into the specific context of UHI tools and the broader context of urban planning theory. Community participation was thus interpreted as a key factor in the development and use of UHI tools in built environment policy and decision-making.

## 1.1.6 Theory of Change

A theory of change (ToC) is a guiding framework for evaluation which makes explicit underlying beliefs about how a policy or programme achieves its objectives.^10,11^ The methods and benefits of developing a ToC were popularised through Carol Weiss’s work in evaluation research.^10^ The ToC approach seeks to understand if and how programmes or policy interventions have achieved anticipated goals.^12^ ToC has since been used to evaluate public health initiatives, among many other policy areas.^10,13,14^

Popay et al. recommend developing a theory of change (ToC) to inform the conduct of a narrative synthesis and to describe findings.^6^ The ToC in this study was developed iteratively from the early scoping review and during the process of conducting the narrative synthesis. The ToC elaborates ‘what worked’ (inputs and activities, leading to outputs and outcomes), in ‘what circumstances’ (UHI tool development approach and wider context), and ‘for whom’ (residents, local government and other stakeholders).

A ToC can be developed through a range of methods, including: a participatory process with stakeholders, document review, and interviews.^10,11^ In selecting our method to develop a ToC we reviewed guidance from the literature and conducted a rapid literature review of other relevant ToCs.^10,11,14^ Allen et al.’s ToC was analogous to developing a ToC for the use and value of UHI tools.^15^ Allen et al. created a ToC for decision support systems for rabbit management, a complex environmental challenge in Australia. Similarly to UHI tools, they argued that decision support systems aim to support practitioners with complex decision-making, yet they remain underutilised and stakeholder participation has been advocated to increase their use (ibid). They proposed ToC as a process to inform the design, use and evaluation of two decision support systems. Their study demonstrated the value of a ToC approach to describe how UHI tools influence complex policy and decision-making contexts where multiple stakeholders are involved.

The process of developing a ToC was iterative and used conceptual mapping and coding qualitative data (in Nvivo) for the thematic analysis. Figure 1 shows an early version of the ToC. Early versions of the ToC were drawn and re-drawn informally and aided exploration of the use and value of UHI tools for different urban actors (in other words, what worked, for whom, and in which circumstances). Figure 1 shows how the researchers interpreted and made sense of two stages of UHI tools (development and application) which can be either expert-led or participatory (on a spectrum). The figure also shows how certain processes were understood as iterative or cyclical through the use of a circular arrow.

**Figure 1 Early version of theory of change used to guide the narrative synthesis analysis**

As previously stated, we used data coded during the narrative synthesis of systematic review studies to develop the ToC. We identified varied uses and benefits (outcomes) of UHI tools based on how they were developed, either through a participatory or an expert-led process. These were identified as two key ‘approaches’ for the ToC. The elements in the ToC (i.e. context, inputs, activities, outputs and outcomes) were identified through data coded in Nvivo from the thematic analysis, shown in Table 2. Data coded as barriers and motivation were translated to context. The facilitators and barriers to UHI tool development and application were translated into inputs. The descriptions of how UHI tools were developed were translated to activities. The uses and benefits of using UHI tools naturally aligned with outputs and outcomes respectively. Differentiation between outputs and outcomes was based on whether they were achieved in the short to mid-term (outputs) and long-term (outcomes). We completed a checklist created by Breuer et al. to increase transparency when reporting a ToC (Table 3).^14^

**Table 2 Nvivo codes and sub-codes from which theory of change (ToC) elements were derived**

| **ToC element** | **Nvivo codes** | **Nvivo sub-codes** |
| --- | --- | --- |
| Inputs | Facilitators | Data quality  Linked to other monitoring requirements  Partnership working  Presentation of information  Scale of data is local  Tied to policy |
|  | Barriers | N/A |
| Activities | Indicator development process | Evidence-based  Experts  Multi-stakeholder co-production  Public health leading community group |
|  | Community participation | Capacity building  Community actions to avoid harms (sub-code: Power(less))  Community used indicators  Stakeholder relations |
| Outputs and outcomes | Uses or benefits of UHI tool | Benchmarking  Capacity building in local government  Collaboration  Communication  Create awareness of urban health issues  Definition of urban health concept  Engage politicians  Engage public or change behaviour  Funding allocation  Highlight community needs  Identification of local issues  Improve or protect environment  Informed decisions (sub code: evaluate development proposals)  Informed policy development  Justification of policy or decision  Monitoring  Ownership  Performance management  Prioritisation of policy areas  Reduce inequalities  Target resources or policies  Transparency (sub-code: gaining trust) |
|  | Community participation | See above |
| Context | Barriers | N/A |
|  | Motivation | N/A |

**Table 3 Completed Theory of Change checklist from Breuer et al.**^14^

| **ToC Checklist by Breuer et al.** ^14^ | **Responses for this study** |
| --- | --- |
| 1. Is the ToC approach defined?   1. Is a definition of ToC given by the authors? 2. Do the authors explain their reasons for using a ToC approach? | 1. Provided definitions from ^10,11,14^ 2. Justified producing a ToC due to recommendation by Popay et al. guidance, a related example from the literature (Allen et al.), and findings from Breuer et al. systematic review of ToC in public health intervention evaluation. |
| 2. Is the ToC development process described?   1. Are the methods used to develop the ToC, such as stakeholder meetings and interviews, document reviews, programme observation, existing conceptual frameworks or published research, described? 2. Where stakeholders are involved, is it clear how many stakeholders participated, what their role is in relation to the intervention, how they were consulted (e.g. number of interviews, focus groups, ToC workshops) and the extent to which the consultations were participatory? 3. Is the method used to compile the data into a ToC described? (including how disagreements between stakeholders were resolved) 4. Is the extent to which stakeholders were able to validate the resultant ToC and were owners of the final product described? | 1. The ToC was developed using the process of narrative synthesis (of systematic review studies) and supported by findings from the wider literature. 2. Stakeholders were not involved. 3. The methods section explains that data from the systematic review informed the ToC development. 4. The desirability of future stakeholder validation is covered in the Discussion section. |
| 3. Is the resultant ToC (or a summary thereof) depicted in a diagrammatic form and does it include?   1. The long-term outcome or impact of the intervention 2. The anticipated short and medium term outcomes and the process of change 3. The intervention components which happen at different stages of the pathway 4. The context of the intervention 5. Assumptions about how change would occur 6. Additional ToC elements such as indicators, supporting research evidence, beneficiaries, actors in the context, sphere of influence and timelines where relevant. | The ToC is described through a diagram and a table.   1. Yes, both include reference and/or detail of the long-term outcome and desired impact. 2. Yes, both include reference and/or detail of the short and medium-term outcome and the process of change. 3. The intervention components are described in relation to the indicator development and application process. 4. The context is described in both diagram and table. 5. Assumptions are described regarding how change would occur in terms of the generation of new knowledge, collaborations and actions. 6. Additional elements include the relevant actors and supporting research evidence. |
| 4. Is the process of intervention development from the ToC described?   1. Are the methods of how interventions were refined from the ToC to something which can be implemented described? (For example, further stakeholder workshops, interviews, systematic literature reviews) | Yes, further testing of the ToC is described in the Discussion section, including remarks on the value of developing a ToC to inform future UHI tool projects. |
| 5. Is the way in which the ToC was used to develop and implement the evaluation described?   1. Are evaluation research questions generated from the ToC? 2. Is the role of ToC in the design, plan or conduct of the evaluation clear? 3. Does the paper describe the extent to which the key elements described in the ToC were measured in the evaluation (i.e. impact, short and medium term outcomes and the process of change, context, assumptions and the intervention)? 4. Does the paper describe whether and how process indicators were used to improve the quality of the intervention? 5. Is the role of the ToC in the analysis of the results of the evaluation clear? 6. Is the role of ToC in the interpretation of the results of the evaluation described? (including the breakdown of programme theory, unanticipated outcomes and causation including the strength and direction of causal relationships) | 1. The ToC was developed iteratively during the narrative synthesis and early examples informed the systematic review. The review findings and ToC were developed together. 2. Yes, the role of the ToC in the overall study is described in chapters 1, 3 and 5. 3. Yes, there is a description of the ToC elements being derived from the narrative synthesis. 4. Process indicators would not have been appropriate in this context. 5. Yes, the role of the ToC is clearly explained as both informing the narrative synthesis and being a result of the narrative synthesis. 6. The ToC is described in the Discussion section alongside other results and we state that the narrative synthesis and ToC were developed together. |

## 1.1.7 Expert-led and participatory development processes

We used information provided by UHI tool producers (i.e. from studies reported in Part A and one more recently published study) and studies reporting the use of UHI tools (i.e. Part B studies) to extract data on how UHI tools were developed. The Part B studies were coded in Nvivo using the following codes about UHI tool development: indicator development process; evidence-based; experts; multi-stakeholder co-production; public health leading community group; and community participation. We supplemented this information with data from Part A studies (and wider literature) if further UHI tool development details were available. The extracts in Table 4 show the data that we used to categorise tools as either ‘expert-led’ or ‘participatory’. We also briefly justify our classification in Table 4 by summarising the key features of the UHI tool development process that relate to either ‘expert-led’ or ‘participatory’ approaches.

**Table 4 Excerpts from literature about included UHI tools regarding their development process used in classification as ‘expert-led’ or ‘participatory’ with justification for such classification.**

| **Development** | **Data extract and source** | **Justification for classification as expert-led or participatory** |
| --- | --- | --- |
| **Expert-led** | | |
| (Bristol) Quality of Life Indicators | ‘In response to [United Nations Conference on the Environment Development in 1992] in 1995, Bristol City Council produced its ﬁrst “State of the Local Environment Report” which included Bristol’s own QoL indicators – then known to Bristol as “Sustainability Indicators”. (…) The sustainability indicators were updated annually and were included, on government advice in 1997, in Bristol’s Local Agenda 21 strategy. These liveability indicators were based on the top eight issues in Bristol, including street litter, grafﬁti, dog fouling, air quality and trafﬁc pollution, environmental noise, state of local rivers and streams, appearance of streets and public spaces and quality of parks and green spaces. (…) In 2000/2001 a new duty was placed on Local Authorities to develop a Community Strategy, the aim of which was to promote social, economic and environmental wellbeing. This meant Bristol was in a strong position to build its Community Strategy on the existing LA21 strategy, with QoL indicators as its monitoring framework. The local government auditor and innovator, the Audit Commission then began a pilot in 2001 to develop a national set of QoL indicators and because of the history and strength of it’s [sic] work, Bristol became a good-practice example of how to use QoL indicators. (…) The pilot process encouraged the use of new indicators and saw Bristol expand its QoL work into in-depth survey work, where in 2001 the ﬁrst “Quality of Life in your Neighbourhood Survey” took place, randomly selecting 4,000 residents to survey on QoL issues.’^16^ | The city council developed the indicators based on the city’s top issues and later used residents’ opinions via a survey as indicator data. |
| Community Indicators Victoria | ‘The development and consensus of a conceptual framework that guides the indicators collected in a community indicator system is a large and complex task. The process will determine what factors are common to understandings of community wellbeing and societal progress and indicators that reflect this definition. Development of CIV’s framework was completed as an initial collaborative project across an 18 month period from 2005 to 2006 (Wiseman et al. 2006). Funded by the Victorian Health Promotion Foundation (VicHealth), the project capitalized on the expertise and ideas of many representatives from state government departments, local government representatives from 35 municipalities, the Australian Bureau of Statistics, the Municipal Association of Victoria, the Victorian Local Government Association, the Victorian Council of Social Services, academics, written submissions and attendees at public consultation forums held across Victoria. This approach balanced top-down and bottom-up contributions to the framework’s development.’^17^  ‘The outcome of the pilot project was a comprehensive framework of community wellbeing including a broad range of indicators to identify and measure wellbeing according to the five broad domains of social, economic, environ- mental, democratic and cultural wellbeing. (…) These domains were agreed upon after extensive consultation and collaboration over an 18-month period with numerous representatives from academia, community organizations, local government and state government departments.’^18^ | A participatory process was used to determine the conceptual framework and domains, however indicators were selected by the expert team. |
| Places Rated Almanac | ‘We have conversed at length with both Boyer and Savageau [the authors of *Places Rated Almanac*]. Boyer noted that after they conceived the idea of Places Rated he began a systematic search of the literature in an attempt to choose indicators. (Both were former book editors and were familiar with the works of many well-known social scientists.) He read the classics in social indicators but finally decided the whole area was unnecessarily complex for their needs. The team developed the concepts and the measures using common sense, and the sales of the book provide evidence that they have struck a responsive chord among readers.’^19^ | The authors developed the indicators independently using literature review and ‘common sense’. |
| (Seattle) Healthy Living Assessment (HLA) | ‘In 2010, the City of Seattle received a Communities Putting Prevention to Work (CPPW) Healthy Eating Active Living grant from Public Health – Seattle & King County to incorporate health into neighborhood planning. Between October 2010 and December 2011, an interdepartmental working group with representatives from DPD, SDOT, and DON worked with Healthy Communities Consulting to develop the Healthy Living Assessment (HLA) tool and incorporate it into the neighborhood plan update process. In 2011, the HLA tool was piloted in two neighborhoods undergoing neighborhood plan updates, Rainier Beach and Broadview‐Bitter Lake‐Haller Lake.’^20^ | A cross-departmental city government working group developed the assessment tool with an independent consulting company. Community views are then gathered using the pre-set list of indicators. |
| **Participatory** | | |
| Core Environmental Health Indicators in Lucknow and Calcutta | ‘Techniques were used to discuss environmental problems and to then develop indicators. Focus-group discussions and Participatory Rapid Appraisal techniques, such as disease seasonality mapping and drawing for children, were also undertaken. (…) …household interviews were held to initiate the process of indicator development. On the basis of these interviews, mapping and photography were used to explore local perceptions of the environment. The results of these various methods, such as the photographs and maps, were then used to stimulate discussion in larger group meetings, in which the concept of indicators was introduced. Residents were asked to imagine that they were service planners and to decide which important environmental factors they would want to monitor. (…) When community-based indicators had been developed in the study sites, workshops were held between residents and service providers. These fora were used as an opportunity to discuss the proposed indicators, as well as the general service situation.’^21^ | Detailed household interviews and focus group activities were used to select indicators. Residents and service providers also interacted to select indicators and discuss local service delivery problems. |
| Flemish City Monitor | ‘…the City Monitor was being developed with the participation of about 200 experts, coming from city governments and other administrations, civil society and academic world. It is our argument that the participatory approach fosters the use of community indicators and generates interesting side effects. (…) In the first two phases hundreds of ‘experts’ were involved. Those ‘experts’ participated in the development of these indicators, on the basis of their involvement in a better quality of life in Flemish cities. They came from different levels in government circles, the civil society and academia. (…) The expert meetings were always organised per activity domain and were attended by an average of 15 experts from the major cities as well as the Flemish administration, the civil society and the academic world. In this way, hundreds of experts were consulted. During these meetings, intentions were added, fine-tuned and removed and this resulted in the definite shape and content of the vision matrix. In all those meetings a high degree of consensus was reached.’^22^ | Indicators were co-developed with civil servants, civil society and academics. |
| Richmond Health and Wellness Element Indicators | ‘In Richmond, community groups and the city government drafted a Health and Wellness Element—or a development and policy blueprint—as part of the city’s General Plan Update. Community-based organizations also led their own processes to collect data for and draft health equity indicators. (…) …the indicator process emerged from ongoing community organizing and land use planning, and included community-based organizations and the city and county health department. Community priorities were highlighted through a process called ‘‘Measuring What Matters’’ where over ten different community-based organizations identified priority issues, chose indicators, collected and analyzed data, and published a comprehensive report that included quantitative and qualitative information. At the same time, the city organized a participatory process to draft and implement the Health and Wellness Element, which included a set of goals and metrics aimed at promoting and monitoring progress on population health [33]. In order to track and monitor indicators on an ongoing basis, the Richmond Health Equity Partnership (http:// richmondhealth.org) was established in 2012 and includes representatives from the city, county health department, school district, and a host of community-based organisations.’^23^ | City/county representatives worked with community-based organisations. The latter created their own process to develop indicators, including data collection and analysis. |
| Richmond Health Equity Indicators (aka Healthy City Diamonds) | ‘…a Technical Advisory Committee was established to help draft the CHWE [Community Health and Wellness Element] that included both community activists and public health scientists. (…) The RHEP was an extension of the CHWE technical advisory committee and pilot implementation initiatives but included additional departments from both city and county governments, as well as representatives from the local school district and a host of non-proﬁt and community-based organizations. (…) A ﬁrst stage in coproducing the HiAP strategy in Richmond was to work with residents to develop a framework for identifying the key drivers of health inequities and the ways local policy might promote greater health equity. (…) Some community activists noted that they were also involved in the drafting of a screening tool by the California Ofﬁce of Environmental Health Hazard Assessment (OEHHA) called “EnviroScreen,” that was to become the nation’s ﬁrst comprehensive methodology for identifying multiple environmental, social, and health vulnerabilities at the community scale (http://oehha.ca.gov/ej/ces2.html), and that a similar framework could be applied in Richmond. (…) ‘Building on the idea of cumulative environmental stressors raised by the CalEnviroScreen methodology, public health professionals participating in the workshops shared what was called the “cumulative toxic stressors model. (…) Participatory workshops with a range of stakeholders, from community to city staff, were also crucial for generating policy solutions and transforming the governance relationships between the city and its residents.’^24^ | Community activists worked with public health scientists and city staff determine indicators and policy. |
| San Francisco Indicator Project (SFIP) | ‘In 2007 the San Francisco Department of Public Health, with broad public input, developed a comprehensive system of neighborhood indicators to inform, influence, and monitor decisions made by the Department of City Planning and other community development institutions. (…) The department vetted the candidate measures with community and public agency stakeholders as well as additional national experts and advisers and ultimately decided on a final list of indicators.’^25^  ‘…SFDPH convened and facilitated a multi-stakeholder Community Council of organizations to implement the Eastern Neighborhoods Community Health Impact Assessment (ENCHIA). (…) SFDPH would staff the assessment, gather data, and conduct research in response to the interests and critiques of the Council. All decisions, recommendations, and outcomes would result from Council deliberation. (…) SFDPH invited more than 40 organizations affected by the Eastern Neighborhoods Community Planning process to join the Community Council; 25 groups chose to participate. Members of the ENCHIA Community Council represented broad interests, including community planning and design, economic and neighborhood development, environmental justice, homelessness, open space, housing, sustainable transportation, food systems, childcare and childhood development advocates, small businesses, and low-wage and union workers. Nonproﬁt and private developers, property owners, and architects represented the development community. City agencies also participated in Council meetings, answering questions regarding agency programs and policies, and sharing agency-speciﬁc planning and assessment data. The Center for Collaborative Policy at California State University, Sacramento, provided consultation on the consensus-building aspect of the process. In addition, a university-based evaluator conducted a process and outcomes evaluation.’^26^ | The city’s department of public health created a Community Council which included community groups, city departments, and private companies (among others). Together, they deliberated what to measure through indicators. |
| Urban Health Equity Indicators for Mathare Informal Settlement | ‘In Mathare, the nongovernmental organization Muungano Support Trust (MuST) has organized residents to survey themselves and document community assets and vulnerabilities in three waves starting in 2007 through 2012 [28,31]. These data have been combined with spatial maps of community assets and hazards and used by MuST in community planning processes focused on specific projects, such as improving housing, water infrastructure, health care access, and community facilities. In 2011, a comprehensive slum redevelopment plan focused on Mathare was drafted by residents, MuST, the University of Nairobi, and University of California, Berkeley, which includes indicators and a process for ongoing monitoring.’^23^ | A nongovernmental organisation worked with residents to determine indicators and gather data. Then working with universities these groups used the indicators to develop a redevelopment plan. |

# 1.2 Relations between characteristics and use of UHI tools

The Part A results showed that 44.1% (64/145) of UHI tools displayed data on maps.^2^ In the qualitative portion of the review, four out of ten UHI tools mapped data; three via interactive maps (San Francisco Indicator Project, Community Indicators Victoria, and Bristol Quality of Life Indicators) and one via a static map (Places Rated Almanac). Presentation of data via maps was rarely mentioned by the studies as being a useful feature of UHI tools, with the exception of Bristol. In Bristol, multiple city stakeholders identified benefits of locally mapped data, including: helping with communication and comparing areas to identify inequalities and build a case for funding, among other benefits (Shepherd and McMahon, 2009). Maps or mapping exercises were used tangentially in Mathare and Seattle. Following the UHI tool project in Mathare, the community derived indicator data were combined with spatial maps about community assets and hazards.^23^ The maps were then used during participatory planning exercises. Rather than presenting data through maps, Seattle’s Healthy Living Assessment tool gathered data through community mapping exercises, where participants identified health-promoting assets and challenges on maps.^20^

Part A identified 53 UHI tools which reported indicators in a composite indicator, or index, comprising 36.6% (53/145) of the UHI tools. The Places Rated Almanac was the only UHI tool included in the qualitative study which produced an index and ranking. Landis and Sawicki summarised the views gathered through interview and survey data about the utility of this UHI tool as follows:

‘Is the Places Rated Almanac useful to planners and public officials? No, not in the slightest sense. For planners, the purposes of comparing places are to learn from other communities and to gain an understanding of how planning can or cannot help reduce crime, improve the quality of health care…[etc.] (…) For planners, the problem with volumes like Places Rated is that we never learn why some places may be superior to others.’^19^

Although Landis and Sawicki found the Places Rated Almanac to affect public opinion and raise the importance of issues to politicians and officers, they did not believe that the indicators were sufficiently robust to inform decision-making on their own. This was not solely attributed to the tool being an index, yet Landis and Sawicki did note challenges with the index methodology and misinterpretations of the rankings by the public and city officials.

# 1.3 UHI tool approaches to address complexity

Corburn and Cohen noted three complexity challenges including: the complexity of urban health (and equity), measuring this system through indicators, and the resultant effect on policy-making.^23^ They proposed using UHI tools in a process of adaptive management (also described elsewhere by Corburn^27^), borrowed from environmental sciences, which involves co-defining problems, co-producing indicators, co-designing policy and co-monitoring and responding to impacts over time. Furthermore, they argued that this approach was well-suited to complex urban health challenges, including equity, because it recognised the failure of linear models and promoted ongoing experimentation and learning in the face of complexity and uncertainty. Their adaptive management UHI tool process took place with residents and other urban stakeholders in Richmond, USA and Mathare, Kenya.^23,24^ However, the stages related to ongoing monitoring and policy adjustments were not reported in either of these settings. Corburn and Cohen reported that this approach was still new and required further adoption and evaluation to determine its effectiveness.^23^

Van Assche et al. proposed that indicators could support the complexity of policy and decision-making in cities by providing local knowledge and feeding policy debate.^22^ They recognised that ‘a complex urban policy debate weighs heavily on decision making in public policies’ and that indicators could help reduce uncertainty in this process.^22^ They used uncertainty and complexity as ‘theoretical building blocks’ for the creation of the UHI tool, alongside a ‘value base’ which consisted of a ‘normative framework on liveable and sustainable urban development...’^22^ By developing indicators within this theoretical and normative framework, Van Assche and colleagues intended to provide decision-makers with indicators which represented local knowledge to inform policy debates. In their estimation, the Flemish City Monitor had not been widely adopted by city officials and politicians because they did not support the monitoring and city comparison elements of the UHI tool. They argued that ‘carefully selected, relevant and interpretable indicators can contribute, but the real issue is likely to be about the integration of needs in all policy domains.’^22^ In other words, UHI tools could help with complex challenges if they succeed in influencing multiple policy domains.

# 1.4 Completed quality appraisal checklists

A National Institute for Health and Care Excellence Quality Appraisal Checklist was completed for each study included in the systematic review narrative synthesis.^3^ Table 5 shows the full checklist and the subsequent tables (Tables 5-14) are an abridged version that contain the appraisal comments but reduce the example text for each question.

**Table 5 Completed quality appraisal checklist for Bhatia**^25^

| **Study identification:** Include author, title, reference, year of publication | Bhatia, R., 2014. Case Study: San Francisco’s Use Of Neighborhood Indicators To Encourage Healthy Urban Development. Health Affairs 33, 1914–22. | |
| --- | --- | --- |
| **Guidance [STUDY] topic:** Use of San Francisco Indicators Project | **Key research question/aim:** ‘This article provides a case study of San Francisco’s experience with neighborhood indicators, describing applications of the indicators to community design and development. The account is based on my experience and observations as the director of the design, development and application of the indicators in San Francisco between 2007 and 2013.’^25^ | |
| **Checklist completed by:** | Helen Pineo | |
| **Theoretical approach** | | |
| **1. Is a qualitative approach appropriate?**  For example:   - Does the research question seek to understand processes or structures, or illuminate subjective experiences or meanings? - Could a quantitative approach better have addressed the research question? | Appropriate | Comments:  The case study explains processes and could not have been conducted with a quantitative approach. |
| **2. Is the study clear in what it seeks to do?**  For example:   - Is the purpose of the study discussed – aims/objectives/ research question/s? - Is there adequate/appropriate reference to the literature? - Are underpinning values/assumptions/theory discussed? | Clear | Comments:  This is a case study without a research question stated. Literature is referenced. Underpinning values, etc. not discussed. |
| **Study design** | | |
| **3. How defensible/rigorous is the research design/methodology?**  For example:   - Is the design appropriate to the research question? - Is a rationale given for using a qualitative approach? - Are there clear accounts of the rationale/justification for the sampling, data collection and data analysis techniques used? - Is the selection of cases/sampling strategy theoretically justified? | Appropriate | Comments: This is a case study and is therefore subjective in nature. It states one person’s perceptions of the example. However, it provides a detailed account of a process occurring over several years and is therefore amenable to a case study design. |
| **Data collection** | | |
| **4. How well was the data collection carried out?**  For example:   - Are the data collection methods clearly described? - Were the appropriate data collected to address the research question? - Was the data collection and record keeping systematic? | Not described | Comments:  Although data collection is not described, this is a first-hand account of a process led by the author. |
| **Trustworthiness** | | |
| **5. Is the role of the researcher clearly described?**  For example:   - Has the relationship between the researcher and the participants been adequately considered? - Does the paper describe how the research was explained and presented to the participants? | Clearly described | Comments:  The author is potentially biased based on his role leading the development/application of the indicators being described. |
| **6. Is the context clearly described?**  For example:   - Are the characteristics of the participants and settings clearly defined? - Were observations made in a sufficient variety of circumstances - Was context bias considered | Minimally described | Comments:  The author reports the circumstances of the use of the indicators on the Eastern Neighborhoods planning process and several other examples. Bias not discussed. |
| **7. Were the methods reliable?**  For example:   - Was data collected by more than 1 method? - Is there justification for triangulation, or for not triangulating? - Do the methods investigate what they claim to? | Not described | Comments: |
| **Analysis** | | |
| **8. Is the data analysis sufficiently rigorous?**  For example:   - Is the procedure explicit – i.e. is it clear how the data was analysed to arrive at the results? - How systematic is the analysis, is the procedure reliable/dependable? - Is it clear how the themes and concepts were derived from the data? | Not described | Comments: |
| **9. Is the data 'rich'?**  For example:   - How well are the contexts of the data described? - Has the diversity of perspective and content been explored? - How well has the detail and depth been demonstrated? - Are responses compared and contrasted across groups/ sites? | Appropriate | Comments:  The case study itself provides a rich account of the circumstances surrounding the UHI tool. Diverse perspectives are considered. |
| **10. Is the analysis reliable?**  For example:   - Did more than 1 researcher theme and code transcripts/data? - If so, how were differences resolved? - Did participants feedback on the transcripts/data if possible and relevant? - Were negative/discrepant results addressed or ignored? | Not reported | Comments:  The case study was written by one person with no explicit discussion of data collection and analysis. |
| **11. Are the findings convincing?**  For example:   - Are the findings clearly presented? - Are the findings internally coherent? - Are extracts from the original data included? - Are the data appropriately referenced? - Is the reporting clear and coherent? | Appropriate | Comments:  The case study description is clear and coherent. |
| **12. Are the findings relevant to the aims of the study?** | Yes | Comments: |
| **13. Conclusions**  For example:   - How clear are the links between data, interpretation and conclusions? - Are the conclusions plausible and coherent? - Have alternative explanations been explored and discounted? - Does this enhance understanding of the research topic? - Are the implications of the research clearly defined?   **Is there adequate discussion of any limitations encountered?** |  | Comments:  This case study provides one person’s perspective, without discussion of methods (data collection, analysis, etc.). The conclusions are plausible in relation to other similar literature. The case study enhances understanding of the research topic.  There is no discussion of limitations. |
| **Ethics** | | |
| **14. How clear and coherent is the reporting of ethics?**  For example:   - Have ethical issues been taken into consideration? - Are they adequately discussed e.g. do they address consent and anonymity? - Have the consequences of the research been considered i.e. raising expectations, changing behaviour? - Was the study approved by an ethics committee? | Not reported | Comments: |
| **Overall assessment**  **As far as can be ascertained from the paper, how well was the study conducted? (see guidance notes)**  ++  +  − | +/- | Comments:  Although methods were not reported, this case study provides a rich account of the San Francisco Indicator Project development by the person who led the work. |

**Table 6 Completed quality appraisal checklist for Corburn et al.**^24^

| **Study identification:** Include author, title, reference, year of publication | Corburn, J., Curl, S., Arredondo, G., Malagon, J., 2014. Health in All Urban Policy: City Services through the Prism of Health. J Urban Health 91, 623–636. | |
| --- | --- | --- |
| **Guidance [STUDY] topic:** Case study of HiAP approach in Richmond, CA and the use of health equity indicators within this process | **Key research question/**  **aim:** ‘In this paper, we describe and analyze the emergence and development of HiAP in Richmond, California, over the past 4 years. …Using these data, we reveal the conceptual frames, practical strategies, and evaluation evidence that contributed to an urban Health in All Policies practice explicitly focused on addressing health equity.’^24^ | |
| **Checklist completed by:** | Helen Pineo | |
| **Theoretical approach** | | |
| **1. Is a qualitative approach appropriate?** | Appropriate | Comments:  Explains processes, subjective experiences or meanings. |
| **2. Is the study clear in what it seeks to do?** | Clear | Comments:  The aim of the research is briefly described (no research question clearly stated). Literature is referenced. Underpinning values, etc. of the research method are not discussed. |
| **Study design** | | |
| **3. How defensible/rigorous is the research design/methodology?** | Difficult to judge due to lack of details | Comments:  The case study is appropriate to provide a rich account of the HiAP process in Richmond. Authors describe data collected using participant observation, interviews, and document review. Sampling, detailed data collection methods and data analysis are not reported. |
| **Data collection** | | |
| **4. How well was the data collection carried out?** | Appropriate | Comments:  Authors describe data collected using participant observation, interviews, and document review. Data were collected over a 4 year period. |
| **Trustworthiness** | | |
| **5. Is the role of the researcher clearly described?** | Not described | Comments:  The role of the researchers in the HiAP project is not clearly described. |
| **6. Is the context clearly described?** | Minimally described | Comments:  The authors report the context. Observation details and context bias were not discussed. |
| **7. Were the methods reliable?** | Appropriate data collection methods were applied, although analysis was not discussed. | Comments:  Data were collected using multiple methods. Analysis was not described. |
| **Analysis** | | |
| **8. Is the data analysis sufficiently rigorous?** | Not described | Comments: |
| **9. Is the data 'rich'?** | Yes | Comments:  The case study describes a rich analysis of HiAP process in Richmond. There are no quotes from interview data. |
| **10. Is the analysis reliable?** | Not reported | Comments: |
| **11. Are the findings convincing?** | Findings are clearly described. | Comments:  The findings are plausible in relation to the wider literature. No extracts or references to data. |
| **12. Are the findings relevant to the aims of the study?** | Yes | Comments: |
| **13. Conclusions**  **Is there adequate discussion of any limitations encountered?** |  | Comments:  This case study approach is well-suited to describe the process of HiAP in Richmond and does enhance understanding of the research topic.  Regarding limitations, these are not reported. |
| **Ethics** | | |
| **14. How clear and coherent is the reporting of ethics?** | Not reported | Comments: |
| **Overall assessment**  **As far as can be ascertained from the paper, how well was the study conducted? (see guidance notes)**  ++  +  − | +/- | Comments:  The case study provides some detail on methods but does not describe analysis or limitations. Overall, it provides useful research findings on a topic that is not widely researched. |

**Table 7 Completed quality appraisal checklist for Corburn and Cohen**^23^

| **Study identification:** | Corburn, J., Cohen, A.K., 2012. Why We Need Urban Health Equity Indicators: Integrating Science, Policy, and Community. PLOS Med 9, e1001285. | |
| --- | --- | --- |
| **Guidance [STUDY] topic:** Use of indicators in Richmond, California and Nairobi, Kenya | **Key research question/aim:**  ‘In this paper, we briefly outline an approach for promoting greater urban health equity through the drafting and monitoring of indicators. We draw examples from the cities of Richmond, California, and Nairobi, Kenya.’^23^ | |
| **Checklist completed by:** | Helen Pineo | |
| **Theoretical approach** | | |
| **1. Is a qualitative approach appropriate?** | Appropriate | Comments:  The research explains processes and a qualitative approach is most appropriate. |
| **2. Is the study clear in what it seeks to do?** | Clear | Comments:  This is a short case study example used to illustrate points in a larger paper without a research question stated. Literature is referenced. Underpinning values, etc. not discussed. |
| **Study design** | | |
| **3. How defensible/rigorous is the research design/methodology?** | Not a study | Comments:  Describes the experience of the authors: ‘Drawing from our collaborative work on healthy urban governance and the drafting of health equity indicators in Richmond, California, and the Mathare Valley…’ (p.2) |
| **Data collection** | | |
| **4. How well was the data collection carried out?** | Not described | Comments: |
| **Trustworthiness** | | |
| **5. Is the role of the researcher clearly described?** | Not described | Comments:  The researchers were involved in developing the indicators but it is not clearly described. |
| **6. Is the context clearly described?** | Minimally described | Comments:  The authors briefly report the circumstances of the use of the indicators. Context bias not discussed. |
| **7. Were the methods reliable?** | Not described | Comments: |
| **Analysis** | | |
| **8. Is the data analysis sufficiently rigorous?** | Not described | Comments: |
| **9. Is the data 'rich'?** | No data is explicitly described | Comments:  The account of these examples is brief and would probably not be described as ‘rich’. |
| **10. Is the analysis reliable?** | Not reported | Comments: |
| **11. Are the findings convincing?** | Minimally | Comments:  The descriptions of the use of indicators are very brief. |
| **12. Are the findings relevant to the aims of the study?** | Not described | Comments: |
| **13. Conclusions**  **Is there adequate discussion of any limitations encountered?** |  | Comments:  These short case study examples provide the authors’ perspectives, without discussion of methods (data collection, analysis, etc.) and therefore there are several limitations. However the findings do enhance understanding of the research topic.  There are no discussions of limitations. |
| **Ethics** | | |
| **14. How clear and coherent is the reporting of ethics?** | Not reported | Comments: |
| **Overall assessment**  **As far as can be ascertained from the paper, how well was the study conducted? (see guidance notes)**  ++  +  − |  | Comments:  These short case study descriptions are not presented as a study and therefore no score is given. |

**Table 8 Completed quality appraisal checklist for Farhang et al.**^26^

| **Study identification:** | Farhang, L., Bhatia, R., Scully, C.C., Corburn, J., Gaydos, M., Malekafzali, S., 2008. Creating Tools for Healthy Development: Case Study of San Franciscoʼs Eastern Neighborhoods Community Health Impact Assessment. Journal of Public Health Management and Practice 14, 255–265. | |
| --- | --- | --- |
| **Guidance [STUDY] topic:** Case study of Eastern Neighborhoods Community Health Impact Assessment and use of indicators ‘Healthy Development Measurement Tool’ | **Key research question/**  **aim:** ‘This case study describes the 18-month ENCHIA process, key outcomes, and lessons learned. The case study also provides an overview of the Healthy Development Measurement Tool and examples of its first applications to urban planning.’^26^ | |
| **Checklist completed by:** | Helen Pineo | |
| **Theoretical approach** | | |
| **1. Is a qualitative approach appropriate?** | Appropriate | Comments:  Explains processes, subjective experiences or meanings and therefore qualitative approach is appropriate. |
| **2. Is the study clear in what it seeks to do?** | Clear | Comments:  The aim of the case study is described (no objectives/ research question stated). Literature is referenced. Underpinning values, etc. of the research method are not discussed. |
| **Study design** | | |
| **3. How defensible/rigorous is the research design/methodology?** | Difficult to judge due to lack of details | Comments:  No methods are described however the case study approach is appropriate to provide a detailed account of the development of the San Francisco Indicators Project (SFIP). |
| **Data collection** | | |
| **4. How well was the data collection carried out?** | Not described | Comments: |
| **Trustworthiness** | | |
| **5. Is the role of the researcher clearly described?** | Not described | Comments:  The authors include individuals who were creating the indicators and health impact assessment (HIA) being discussed as well as external authors who may also have been involved (not stated). |
| **6. Is the context clearly described?** | Minimally | Comments:  The stakeholders involved (not described as participants) were clearly described. Observations were clearly made in a wide variety of circumstances. Context bias was not discussed. |
| **7. Were the methods reliable?** | Unknown | Comments:  The case study does not describe methods. |
| **Analysis** | | |
| **8. Is the data analysis sufficiently rigorous?** | Not described | Comments: |
| **9. Is the data 'rich'?** | Yes | Comments:  The case study does provide rich data about the development of SFIP. |
| **10. Is the analysis reliable?** | Not reported | Comments: |
| **11. Are the findings convincing?** | Minimally | Comments:  Findings are clearly described however lack of data collection and analysis methods descriptions means that judging whether findings are convincing is problematic. |
| **12. Are the findings relevant to the aims of the study?** | Yes | Comments: |
| **13. Conclusions**  **Is there adequate discussion of any limitations encountered?** |  | Comments:  This is a case study approach with unclear methods. The findings are plausible in relation to the wider literature. The findings enhance the understanding of the topic.  There is no discussion of limitations. |
| **Ethics** | | |
| **14. How clear and coherent is the reporting of ethics?** | Not reported | Comments: |
| **Overall assessment**  **As far as can be ascertained from the paper, how well was the study conducted? (see guidance notes)**  ++  +  − | +/- | Comments:  This is a detailed case study but methods are missing. |

**Table 9 Completed quality appraisal checklist for Hunt and Lewin**^21^

| **Study identification:** | Hunt, C., Lewin, S., 2000. Exploring Decision-Making for Environmental Health Services: Perspectives from Four Cities. Reviews on Environmental Health 15, 187–206. | |
| --- | --- | --- |
| **Guidance [STUDY] topic:** Use of environmental health indicators in 4 cities | **Key research question/aim:**  ‘The goal of the study was to explore environmental health service decision-making at the local level. Specifically, the study aimed to compare local authority perceptions of environmental health needs and service status with those of local residents and to explore the space for EHIs [environmental health indicators] in environmental health decision-making.’^21^ | |
| **Checklist completed by:** | Helen Pineo | |
| **Theoretical approach** | | |
| **1. Is a qualitative approach appropriate?** | Appropriate | Comments:  The research seeks to understand process and actors and therefore qualitative research is appropriate. |
| **2. Is the study clear in what it seeks to do?** | Clear | Comments:  States specific aim, reviews literature, refers to other papers which describe the methods in more detail. |
| **Study design** | | |
| **3. How defensible/rigorous is the research design/methodology?** | Rigorous | Comments:  There is a clear explanation of methods and rationale for case study selection (4 cities). |
| **Data collection** | | |
| **4. How well was the data collection carried out?** | Clear and appropriate | Comments:  Data collection is described for different parts of the study and reference is made to other papers by the authors for detailed methods. |
| **Trustworthiness** | | |
| **5. Is the role of the researcher clearly described?** | Partially | Comments:  Paper describes how the research was explained (regarding language issues). The researchers were involved in developing indicators with the case study cities. |
| **6. Is the context clearly described?** | Clear | Comments:  The context of each case is described. Observations were made using different events (attending meetings, running workshops, etc.). |
| **7. Were the methods reliable?** | Yes | Comments:  Data were collected using several methods. Triangulation was used. Methods investigated the relevant aim. |
| **Analysis** | | |
| **8. Is the data analysis sufficiently rigorous?** | Yes | Comments:  Data analysis is clearly described and systematic. Coding was descripted (open coding was used). Themes are explained through interview quote examples. |
| **9. Is the data 'rich'?** | Yes | Comments:  There are descriptions from different perspectives with detail and comparison across cities. The data is rich. |
| **10. Is the analysis reliable?** | Yes | Comments:  It is not clear if multiple researchers coded the data. Triangulation was used with the involvement of researchers from different backgrounds. ‘Negative’ results were discussed. |
| **11. Are the findings convincing?** | Yes | Comments:  Findings are clear, coherent and backed up with extract quotes. Data are referenced and reporting is clear. |
| **12. Are the findings relevant to the aims of the study?** | Relevant | Comments: |
| **13. Conclusions**  **Is there adequate discussion of any limitations encountered?** |  | Comments:  There are clear links between data examples and interpretations. Conclusions are plausible and coherent. The research does increase understanding of this research topic through detailed examples. Implications are discussed.  Limitations are not discussed. |
| **Ethics** | | |
| **14. How clear and coherent is the reporting of ethics?** | Not  reported | Comments:  No discussion of ethics. Possibly reported in the other referenced studies regarding methods. |
| **Overall assessment**  **As far as can be ascertained from the paper, how well was the study conducted? (see guidance notes)**  ++  +  − | ++ | Comments:  Clear methods and rich data and interpretation. |

**Table 10 Completed quality appraisal checklist for Landis and Sawicki**^19^

| **Study identification:** | Landis, J.D., Sawicki, D.S., 1988. A Planner’s Guide to the Places Rated Almanac. Journal of the American Planning Association 54, 336–346. | |
| --- | --- | --- |
| **Guidance [STUDY] topic:** Review of Places Rated Almanac and its value to planners | **Key research question/aim:**  ‘We are concerned first with the appropriateness and reliability of the concepts and measures the Places Rated Almanac uses: how well the place ratings meet the needs and demands of migrating households and individuals who, after all, comprise the primary intended audience for such systems. Second, we are concerned with the extent to which place rating systems evaluate quality of place versus quality of life. Third, we are concerned with how the almanac measures and compares quality of place issues; to address that topic, we examine the types of categories and components the publication includes. Fourth, we are concerned with how the results of such schemes do or do not affect the conduct of local planning and policy making.’ ^19^ | |
| **Checklist completed by:** | Helen Pineo | |
| **Theoretical approach** | | |
| **1. Is a qualitative approach appropriate?** | Appropriate | Comments:  The study used a quantitative survey of actors views (conducted via interviews) on the Places Rated Almanac, therefore this is quantitative social research. |
| **2. Is the study clear in what it seeks to do?** | Clear | Comments:  States specific aim, reviews literature. Underpinning values, assumptions, theory are not discussed. |
| **Study design** | | |
| **3. How defensible/rigorous is the research design/methodology?** | Appropriate | Comments:  Specifically regarding the use of Places Rated Almanac, the authors surveyed planners in 32 cities (with justification about which planners and which cities). |
| **Data collection** | | |
| **4. How well was the data collection carried out?** | Appropriate | Comments:  Survey methods described in terms of sampling rationale and the method was via interview. |
| **Trustworthiness** | | |
| **5. Is the role of the researcher clearly described?** | Not described | Comments: |
| **6. Is the context clearly described?** | Clear | Comments:  Surveys were in 32 cities. |
| **7. Were the methods reliable?** | Yes | Comments:  Survey was the only method and it did investigate the stated aims. |
| **Analysis** | | |
| **8. Is the data analysis sufficiently rigorous?** | Not described | Comments:  Although the analysis methods are not fully described, the results provide some indication of the analysis which appears to be appropriate. |
| **9. Is the data 'rich'?** | Partially | Comments:  Survey may not have provided depth but gives a good overview of perspectives. |
| **10. Is the analysis reliable?** | Not described | Comments: |
| **11. Are the findings convincing?** | Partially | Comments:  Findings are clear and coherent. Data is not described in detail. |
| **12. Are the findings relevant to the aims of the study?** | Relevant | Comments: |
| **13. Conclusions**  **Is there adequate discussion of any limitations encountered?** | Clear and coherent | Comments:  Useful overview of perceptions of Places Rated Almanac by planning directors in 32 cities.  No discussion of limitations. |
| **Ethics** | | |
| **14. How clear and coherent is the reporting of ethics?** | Not reported | Comments:  No discussion of ethics. |
| **Overall assessment**  **As far as can be ascertained from the paper, how well was the study conducted? (see guidance notes)**  ++  +  − | + | Comments:  Clear methods regarding sampling and survey questions. Clear description of findings. Lack of information on analysis and limitations. |

**Table 11 Completed quality appraisal checklist for Lerman**^20^

| **Study identification** | Lerman, S., 2011. Seattle Healthy Living Assessment: Pilot Implementation Report. | |
| --- | --- | --- |
| **Guidance [STUDY] topic:** Report of development and piloting of Seattle Healthy Living Assessment | **Key research question/aim:**  The report describes the data collection and results of the HLA. It also describes the benefits of using the HLA. | |
| **Checklist completed by:** | Helen Pineo | |
| **Theoretical approach** | | |
| **1. Is a qualitative approach appropriate?** | Appropriate | Comments:  The report explains processes and subjective experiences, although this is not a qualitative study. |
| **2. Is the study clear in what it seeks to do?** | Partially | Comments:  The aim of the report is described (no objectives/ research question stated). Literature and underpinning values are not referenced/discussed. |
| **Study design** | | |
| **3. How defensible/rigorous is the research design/methodology?** | Not relevant. | Comments:  No methods are described – it is a project report not research. |
| **Data collection** | | |
| **4. How well was the data collection carried out?** | Not described | Comments:  The methods for gathering data about the project benefits are not reported. Data collection for the Healthy Living Assessment is reported. |
| **Trustworthiness** | | |
| **5. Is the role of the researcher clearly described?** | Not described | Comments:  The authors include individuals who were creating the indicators and Healthy Living Assessment. |
| **6. Is the context clearly described?** | Context of the project is described | Comments:  No clear participants or observations. Bias was not discussed. |
| **7. Were the methods reliable?** | Unknown | Comments:  The report does not describe methods related to identifying the benefits of the Healthy Living Assessment. |
| **Analysis** | | |
| **8. Is the data analysis sufficiently rigorous?** | Not described | Comments:  The benefits are reported but there is no discussion of how they were collated and reported. |
| **9. Is the data 'rich'?** | No | Comments:  The benefits are described without discussion of different perspectives. There are no quotes from participants or stakeholders. |
| **10. Is the analysis reliable?** | Unknown | Comments:  Analysis was not reported. |
| **11. Are the findings convincing?** | Yes, within limitations. | Comments:  Findings are clearly described but methods are unknown. |
| **12. Are the findings relevant to the aims of the study?** | Yes | Comments: |
| **13. Conclusions**  **Is there adequate discussion of any limitations encountered?** |  | Comments:  This is a project report with unclear methods. However it provides valuable information considering the lack of research on the use of UHI tools. Inadequate discussion of limitations |
| **Ethics** | | |
| **14. How clear and coherent is the reporting of ethics?** | Not reported | Comments: |
| **Overall assessment**  **As far as can be ascertained from the paper, how well was the study conducted? (see guidance notes)**  ++  +  − |  | Comments:  -  This is not reported as a study and therefore the lack of methods and details explaining how findings on the use of the UHI tool were achieved is difficult to assess. |

**Table 12 Completed quality appraisal checklist for Lowe et al.**^28^

| **Study identification:** | Lowe, M., Whitzman, C., Badland, H., Davern, M., Aye, L., Hes, D., Butterworth, I., Giles-Corti, B., 2015. Planning Healthy, Liveable and Sustainable Cities: How Can Indicators Inform Policy? Urban Policy and Research 33, 131–144. | |
| --- | --- | --- |
| **Guidance [STUDY] topic:** Review of liveability indicators and consultation with Melbourne policy-makers on their potential use | **Key research question/aim:**  ‘Our research responds to this challenge of creating liveability indicators that are able to influence policy and practice. Conceptualising liveability through a social determinants of health lens, this article reviews existing liveability indicators and considers how they are utilised. Based on the results of consultations with academics, policymakers from all levels of government, and community and private sector decision-makers in Melbourne, it then considers how indicators could be developed, reported and used to more strongly influence policy and support integrated planning for health, liveability and sustainability.’^28^ | |
| **Checklist completed by:** | Helen Pineo | |
| **Theoretical approach** | | |
| **1. Is a qualitative approach appropriate?** | Appropriate | Comments:  Describes process and actors thus a qualitative approach was appropriate. |
| **2. Is the study clear in what it seeks to do?** | Clear | Comments:  States specific aim, reviews literature. Underpinning values, etc. not discussed. |
| **Study design** | | |
| **3. How defensible/rigorous is the research design/methodology?** | Appropriate | Comments:  Authors used workshops to gather views from policy-makers. Sampling, data collection and analysis is discussed. |
| **Data collection** | | |
| **4. How well was the data collection carried out?** | Appropriately | Comments:  Researchers took notes during workshops and collated these. |
| **Trustworthiness** | | |
| **5. Is the role of the researcher clearly described?** | Not described | Comments:  Unclear if/how this has been described. |
| **6. Is the context clearly described?** | Clear | Comments:  Multiple workshops were used. Bias not discussed. |
| **7. Were the methods reliable?** | Partially | Comments:  Workshop discussions were the only method but it did investigate the stated aim. Triangulation not discussed. |
| **Analysis** | | |
| **8. Is the data analysis sufficiently rigorous?** | Not described | Comments:  Analysis not described. |
| **9. Is the data 'rich'?** | Not sure | Comments:  Results are brief. Perspectives of different people are not reported. |
| **10. Is the analysis reliable?** | Not described | Comments:  Analysis not described. |
| **11. Are the findings convincing?** | Partially | Comments:  Findings are clear and coherent. Data is not described in detail. |
| **12. Are the findings relevant to the aims of the study?** | Relevant | Comments: |
| **13. Conclusions**  **Is there adequate discussion of any limitations encountered?** |  | Comments:  Findings are useful in understanding the research topic. Data is not discussed in detail. Limitations not discussed. |
| **Ethics** | | |
| **14. How clear and coherent is the reporting of ethics?** | Not reported | Comments:  No discussion of ethics. |
| **Overall assessment**  **As far as can be ascertained from the paper, how well was the study conducted? (see guidance notes)**  ++  +  − |  | Comments:  +  Useful study to understand the research topics with some details lacking on analysis. |

**Table 13 Completed quality appraisal checklist for Shepherd and McMahon**^16^

| **Study identification:** | Shepherd, S., McMahon, S., 2009. The Importance of Local Information: Quality of Life Indicators in Bristol, in: Sirgy, P.M.J., Phillips, D.R., Rahtz, P.D.R. (Eds.), Community Quality-of-Life Indicators: Best Cases IV, Community Quality-of Life Indicators. Springer Netherlands, pp. 111–120. doi:10.1007/978-90-481-2243-1_5 | |
| --- | --- | --- |
| **Guidance [STUDY] topic:** Use of QOL indicators in Bristol | **Key research question/aim:**  To demonstrate ‘how important local-level information has been in enabling QoL indicators to become highly effective tools for improving local quality of life’ through ‘decision making, [and] collaboration.’^16^ | |
| **Checklist completed by:** | Helen Pineo | |
| **Theoretical approach** | | |
| **1. Is a qualitative approach appropriate?** | Appropriate | Comments:  Investigates process and actors thus a qualitative approach is appropriate. |
| **2. Is the study clear in what it seeks to do?** | Clear | Comments:  Minimally discussed as described in key research aims above. No reference to literature. No underpinning theory discussed. |
| **Study design** | | |
| **3. How defensible/rigorous is the research design/methodology?** | Inadequately reported | Comments:  No discussion of research design. No rationale. No discussion of participants or sampling. |
| **Data collection** | | |
| **4. How well was the data collection carried out?** | Inadequately reported | Comments:  No discussion of data collection. |
| **Trustworthiness** | | |
| **5. Is the role of the researcher clearly described?** | Not described | Comments:  No discussion of researcher role. |
| **6. Is the context clearly described?** | Unclear | Comments:  Multiple council teams/roles are referenced. No discussion of observations or context bias. |
| **7. Were the methods reliable?** | Not reported | Comments:  No information on methods. |
| **Analysis** | | |
| **8. Is the data analysis sufficiently rigorous?** | Not reported | Comments:  No discussion of data analysis. |
| **9. Is the data 'rich'?** | Not reported | Comments:  There are descriptions of multiple examples across council teams with some quotes. Many perspectives are discussed. |
| **10. Is the analysis reliable?** | Not reported | Comments:  None of the data analysis methods were reported. |
| **11. Are the findings convincing?** | Not reported | Comments:  Findings are clearly presented and internally coherent. Extracts are presented. |
| **12. Are the findings relevant to the aims of the study?** | Relevant | Comments:  Reports multiple built environment policy/decision-making uses of indicators. Reports perceptions of indicators. |
| **13. Conclusions**  **Is there adequate discussion of any limitations encountered?** | Adequate | Comments:  Clear links between data examples and interpretations. Conclusions plausible and coherent. No alternatives discussed. Does increase understanding through detailed examples. Implications mentioned. |
| **Ethics** | | |
| **14. How clear and coherent is the reporting of ethics?** | Not reported | Comments:  No discussion of ethics. |
| **Overall assessment**  **As far as can be ascertained from the paper, how well was the study conducted? (see guidance notes)**  ++  +  − |  | Comments:  +  Useful case study with detailed findings but lacking some information on methods of data collection and analysis. |

**Table 14 Completed quality appraisal checklist for Van Assche et al.**^22^

| **Study identification:** | Van Assche, J.V., Block, T., Reynaert, H., 2010. Can Community Indicators Live Up to Their Expectations? The Case of the Flemish City Monitor for Livable and Sustainable Urban Development. Applied Research Quality Life 5, 341–352. | |
| --- | --- | --- |
| **Guidance [STUDY] topic:** Case study of Flemish City Monitor | **Key research question/aim:**  Stated as questions in the abstract: ‘Does it [Flemish City Monitor] live up to its expectations? And will the vision and indicators on urban sustainability stir up the debate about urban sustainable development?’^22^ | |
| **Checklist completed by:** | Helen Pineo | |
| **Theoretical approach** | | |
| **1. Is a qualitative approach appropriate?** | Appropriate | Comments:  Explains processes, subjective experiences or meanings. |
| **2. Is the study clear in what it seeks to do?** | Clear | Comments:  The aim of the case study is primarily to discuss the development of the Flemish City Monitor. However there is substantive data on the use in local government (with reference to another paper not available in English). Literature referenced. Some discussion of theoretical building blocks of indicators. |
| **Study design** | | |
| **3. How defensible/rigorous is the research design/methodology?** | Unclear. | Comments:  No methods are described – there is reference to another paper (not in English). It is essentially a case study of the authors’ experience developing and using the Flemish City Monitor. |
| **Data collection** | | |
| **4. How well was the data collection carried out?** | Not described | Comments:  No discussion of data collection. |
| **Trustworthiness** | | |
| **5. Is the role of the researcher clearly described?** | Yes | Comments:  The authors were involved in developing the indicators and then handed over the ongoing work to local government. Not clear how the research was described to participants although there is discussion of how they were perceived: ‘after all kinds of suspicions (of policy makers versus academics…’ p.350. |
| **6. Is the context clearly described?** | Minimally | Comments:  Detail of observations is not discussed. The timescales of observation appear to be extensive (at least between 2004 to 2008). Bias was not discussed. |
| **7. Were the methods reliable?** | Not reported | Comments:  The case study does not describe methods (however, there is another paper which is not available in English) |
| **Analysis** | | |
| **8. Is the data analysis sufficiently rigorous?** | Not reported | Comments:  No discussion of data analysis. |
| **9. Is the data 'rich'?** | Yes | Comments:  There are example quotes and a range of perspectives from different people (policy makers and politicians) |
| **10. Is the analysis reliable?** | Not reported | Comments:  None of the data analysis methods were reported. |
| **11. Are the findings convincing?** | Yes | Comments:  Findings are clearly described and convincing.  Findings are clearly presented and internally coherent. Extracts are presented. |
| **12. Are the findings relevant to the aims of the study?** | Relevant | Comments:  Reports multiple built environment policy/decision-making uses of indicators. Reports perceptions of indicators. |
| **13. Conclusions**  **Is there adequate discussion of any limitations encountered?** |  | Comments:  This is a case study approach with unspecified methods. Data are rich and conclusions are plausible. Limitations are not discussed. |
| **Ethics** | | |
| **14. How clear and coherent is the reporting of ethics?** | Not reported | Comments:  No discussion of ethics. |
| **Overall assessment**  **As far as can be ascertained from the paper, how well was the study conducted? (see guidance notes)**  ++  +  − |  | Comments:  +  Useful case study with detailed findings but lacking methods of data collection and analysis. |

# References

1. Pineo H, Glonti K, Rutter H, Zimmermann N, Wilkinson P, Davies M. Characteristics and use of urban health indicator tools by municipal built environment policy and decision-makers: a systematic review protocol. *Syst Rev*. 2017;6:1-6. doi:10.1186/s13643-017-0406-x

2. Pineo H, Glonti K, Rutter H, Zimmermann N, Wilkinson P, Davies M. Urban Health Indicator Tools of the Physical Environment: a Systematic Review. *J Urban Health*. 2018;95(5):613-646. doi:10.1007/s11524-018-0228-8

3. National Institute for Health and Care Excellence. Methods for the development of NICE public health guidelines (Third Edition). 2012. http://www.nice.org.uk/aboutnice/howwework/developingnicepublichealthguidance/ publichealthguidanceprocessandmethodguides/ public_health_guidance_process_and_method_guides.jsp.

4. Fereday J, Muir-Cochrane E. Demonstrating Rigor Using Thematic Analysis: A Hybrid Approach of Inductive and Deductive Coding and Theme Development. *Int J Qual Methods*. 2006;5(1):80-92. doi:10.1177/160940690600500107

5. Braun V, Clarke V. Using thematic analysis in psychology. *Qual Res Psychol*. 2006;3(2):77-101. doi:10.1191/1478088706qp063oa

6. Popay J, Roberts H, Sowden A, et al. Guidance on the conduct of narrative synthesis in systematic reviews. *Prod ESRC Methods Programme Version*. 2006;1:b92.

7. Healey P. *Collaborative Planning: Shaping Places in Fragmented Societies*. Basingstoke: Macmillan; 1997.

8. Innes JEleanor, Booher DE. *Planning with Complexity : An Introduction to Collaborative Rationality for Public Policy*. London: Routledge; 2010.

9. Sterman JD. *Business Dynamics: Systems Thinking and Modeling for a Complex World*. Nachdr. Boston: Irwin/McGraw-Hill; 2000.

10. Weiss CH. *Evaluation: Methods for Studying Programs and Policies*. 2nd ed. Upper Saddle River, N.J.: Prentice Hall; 1998.

11. Morra Imas L, Rist R. *The Road to Results: Designing and Conducting Effective Development Evaluations*. Washington, D.C.: The World Bank; 2009. https://openknowledge.worldbank.org/bitstream/handle/10986/2699/52678.pdf. Accessed July 5, 2018.

12. Bryman Alan. *Social Research Methods*. 2nd ed. Oxford: Oxford University Press; 2004.

13. Connell JP, Kubisch AC. Applying a Theory of Change Approach to the Evaluation of Comprehensive Community Initiatives: Progress, Prospects, and Problems. In: Fullbright-Anderson K, Kubish A, Connell JP, eds. *New Aproaches to Evaluating Community Initiatives: Theory, Measurement, and Analysis, Vol 2.* Washington, D.C.: The Aspen Institute; 1998:15-44.

14. Breuer E, Lee L, De Silva M, Lund C. Using theory of change to design and evaluate public health interventions: a systematic review. *Implement Sci*. 2015;11(63):1-17. doi:10.1186/s13012-016-0422-6

15. Allen W, Cruz J, Warburton B. How Decision Support Systems Can Benefit from a Theory of Change Approach. *Environ Manage*. 2017;59(6):956-965. doi:10.1007/s00267-017-0839-y

16. Shepherd S, McMahon S. The Importance of Local Information: Quality of Life Indicators in Bristol. In: Sirgy PMJ, Phillips DR, Rahtz PDR, eds. *Community Quality-of-Life Indicators: Best Cases IV*. Community Quality-of Life Indicators. Springer Netherlands; 2009:111-120. doi:10.1007/978-90-481-2243-1_5

17. Davern MT, Gunn L, Giles-Corti B, David S. Best Practice Principles for Community Indicator Systems and a Case Study Analysis: How Community Indicators Victoria is Creating Impact and Bridging Policy, Practice and Research. *Soc Indic Res*. 2017;131(2):567-586. doi:10.1007/s11205-016-1259-8

18. Davern MT, West S, Bodenham S, Wiseman J. Community Indicators in Action: Using Indicators as a Tool for Planning and Evaluating the Health and Wellbeing of a Community. In: Sirgy MJ, Phillips R, Rahtz D, eds. *Community Quality-of-Life Indicators: Best Cases V*. Community Quality-of-Life Indicators. Springer Netherlands; 2011:319-338. doi:10.1007/978-94-007-0535-7_14

19. Landis JD, Sawicki DS. A Planner’s Guide to the Places Rated Almanac. *J Am Plann Assoc*. 1988;54(3):336-346. doi:10.1080/01944368808976494

20. Lerman S. Seattle Healthy Living Assessment: Pilot Implementation Report. 2011. http://www.seattle.gov/dpd/cs/groups/pan/@pan/documents/web_informational/dpdd016767.pdf.

21. Hunt C, Lewin S. Exploring Decision-Making for Environmental Health Services: Perspectives from Four Cities. *Rev Environ Health*. 2000;15(1-2):187–206. doi:10.1515/REVEH.2000.15.1-2.187

22. Van Assche J, Block T, Reynaert H. Can Community Indicators Live Up to Their Expectations? The Case of the Flemish City Monitor for Livable and Sustainable Urban Development. *Appl Res Qual Life*. 2010;5(4):341-352. doi:10.1007/s11482-010-9121-7

23. Corburn J, Cohen AK. Why We Need Urban Health Equity Indicators: Integrating Science, Policy, and Community. *PLOS Med*. 2012;9(8):e1001285. doi:10.1371/journal.pmed.1001285

24. Corburn J, Curl S, Arredondo G, Malagon J. Health in All Urban Policy: City Services through the Prism of Health. *J Urban Health*. 2014;91(4):623-636. doi:10.1007/s11524-014-9886-3

25. Bhatia R. Case Study: San Francisco’s Use Of Neighborhood Indicators To Encourage Healthy Urban Development. *Health Aff (Millwood)*. 2014;33(11):1914-1922. doi:http://dx.doi.org.libproxy.ucl.ac.uk/10.1377/hlthaff.2014.0661

26. Farhang L, Bhatia R, Scully CC, Corburn J, Gaydos M, Malekafzali S. Creating Tools for Healthy Development: Case Study of San Franciscoʼs Eastern Neighborhoods Community Health Impact Assessment. *J Public Health Manag Pract*. 2008;14(3):255-265. doi:10.1097/01.PHH.0000316484.72759.7b

27. Corburn J. *Healthy City Planning: From Neighbourhood to National Health Equity*. London ; New York: Routledge; 2013.

28. Lowe M, Whitzman C, Badland H, et al. Planning Healthy, Liveable and Sustainable Cities: How Can Indicators Inform Policy? *Urban Policy Res*. 2015;33(2):131-144. doi:10.1080/08111146.2014.1002606
